# Genome Wide Association Studies Using a New Nonparametric Model Reveal the Genetic Architecture of 17 Agronomic Traits in an Enlarged Maize Association Panel

**DOI:** 10.1371/journal.pgen.1004573

**Published:** 2014-09-11

**Authors:** Ning Yang, Yanli Lu, Xiaohong Yang, Juan Huang, Yang Zhou, Farhan Ali, Weiwei Wen, Jie Liu, Jiansheng Li, Jianbing Yan

**Affiliations:** 1 National Key Laboratory of Crop Genetic Improvement, Huazhong Agricultural University, Wuhan, China; 2 Maize Research Institute, Sichuan Agricultural University, Wenjiang, Sichuan, China; 3 National Maize Improvement Center of China, Beijing Key Laboratory of Crop Genetic Improvement, China Agricultural University, Beijing, China; University of Georgia, United States of America

## Abstract

Association mapping is a powerful approach for dissecting the genetic architecture of complex quantitative traits using high-density SNP markers in maize. Here, we expanded our association panel size from 368 to 513 inbred lines with 0.5 million high quality SNPs using a two-step data-imputation method which combines identity by descent (IBD) based projection and k-nearest neighbor (KNN) algorithm. Genome-wide association studies (GWAS) were carried out for 17 agronomic traits with a panel of 513 inbred lines applying both mixed linear model (MLM) and a new method, the Anderson-Darling (A-D) test. Ten loci for five traits were identified using the MLM method at the Bonferroni-corrected threshold −log_10_ (*P*) >5.74 (α = 1). Many loci ranging from one to 34 loci (107 loci for plant height) were identified for 17 traits using the A-D test at the Bonferroni-corrected threshold −log_10_ (*P*) >7.05 (α = 0.05) using 556809 SNPs. Many known loci and new candidate loci were only observed by the A-D test, a few of which were also detected in independent linkage analysis. This study indicates that combining IBD based projection and KNN algorithm is an efficient imputation method for inferring large missing genotype segments. In addition, we showed that the A-D test is a useful complement for GWAS analysis of complex quantitative traits. Especially for traits with abnormal phenotype distribution, controlled by moderate effect loci or rare variations, the A-D test balances false positives and statistical power. The candidate SNPs and associated genes also provide a rich resource for maize genetics and breeding.

## Author Summary

Genotype imputation has been used widely in the analysis of genome-wide association studies (GWAS) to boost power and fine-map associations. We developed a two-step data imputation method to meet the challenge of large proportion missing genotypes. GWAS have uncovered an extensive genetic architecture of complex quantitative traits using high-density SNP markers in maize in the past few years. Here, GWAS were carried out for 17 agronomic traits with a panel of 513 inbred lines applying both mixed linear model and a new method, the Anderson-Darling (A-D) test. We intend to show that the A-D test is a complement to current GWAS methods, especially for complex quantitative traits controlled by moderate effect loci or rare variations and with abnormal phenotype distribution. In addition, the traits associated QTL identified here provide a rich resource for maize genetics and breeding.

## Introduction

Maize (*Zea mays* L.) is one of the most important food, feed and industrial crops globally. Grown extensively under different climate conditions across the world, maize shows an astonishing amount of phenotypic diversity [1]. Identifying the underlying natural allelic variations for the phenotypic diversity will have immense practical implications in maize molecular breeding for improving nutritional quality, yield potential, and stress tolerance.

With the rapid development of next generation sequencing and high-density marker genotyping techniques, there emerges tremendous interest in using association mapping to identify genes responsible for quantitative variation of complex traits [2]. The use of GWAS has been well demonstrated in model plants such as *Arabidopsis* [3] and rice [4]. In maize, we examined the genetic architecture of maize oil biosynthesis in 368 diverse maize inbred lines with over 1.06 million SNPs obtained from RNA sequencing and DNA array using the GWAS strategy [5]. Despite the great potential that GWAS has to pinpoint genetic polymorphisms underlying agriculturally important traits, false discoveries are a major concern and can be partially attributed to spurious associations caused by population structure and unequal relatedness among individuals in a given panel [6]. A number of statistical approaches have been proposed, among which the mixed linear model (MLM) is one of the popular methods that can eliminate the excess of low *p* values for most traits [6], [7]. However, Zhao et al. [8] performed GWAS using a NAÏVE model in each sub-population and MLM with inferred population structure as a fixed effect in the whole mapping panel of rice, and their results suggested that MLM may lead to false negatives by overcompensating for population structure and relatedness. To improve the MLM, some strategies to best utilize marker data have been proposed [9], [10]. The more we know about the genetics of a trait, the greater our power is to detect the rest of the genetic contribution. The problem is, of course, that we usually do not know what the causal loci are, and methods that try to identify them are prone to over-fitting [11]. Beló et al. [12] adopted the Kolmogorov–Smirnov (KS) test for association analysis in each subpopulation of the mapping panel and an allelic variant of *fad2* associated with increased oleic acid level was successfully identified based on modest density markers. However, detailed instructions for the algorithm were not published. Most current GWAS methods lack the power to detect rare alleles and this has limited the application of GWAS, since rare alleles are common in maize diversity collections [1], [5]. Parametric tests of association are sensitive to SNPs with minor allele frequencies, which can artificially increase association scores. Balancing samples across population subdivisions can homogenize allele frequencies, elevating rates of globally rare variants that are common in certain subdivisions [5].

In this study, 513 diverse maize inbred lines [13], representing tropical/subtropical and temperate germplasm, were genotyped by MaizeSNP50 BeadChip containing 56,110 SNPs [14]. RNA sequencing (RNA-seq) was performed on 368 of these 513 lines and 556,809 high quality SNPs with a minor allelic frequency greater than 0.05 were obtained [5], [15], [16]. Seventeen agronomic traits were systematically phenotyped for the 513 lines under multiple environments and seasons (see Materials and Methods). The objectives of this research were (1) to explore an efficient imputation method to infer missing genotypes for the 145 inbreds that were only genotyped by SNP-chip (low density), not by RNA-seq (high density); (2) to develop a powerful statistical method for GWAS to identify robust QTL for complex agronomic traits in maize; and (3) to methodically analyze the underlying genetic architectures of the 17 agronomic traits in the diverse maize association mapping panel.

## Results

### Phenotypic variation for 17 agronomic traits

A brief description of each trait, its acronym, and evaluation methodology was summarized in Supplementary Table S1. All of the 17 traits in the 513 maize inbred lines were in accordance with a normal distribution (Figure S1A, S2, S3, S4, S5, S6, S7, S8, S9, S10, S11, S12, S13, S14, S15, S16, S17A). But the phenotype of each trait showed distinct differences among four subgroups (Figure S1B, S2, S3, S4, S5, S6, S7, S8, S9, S10, S11, S12, S13, S14, S15, S16, S17B). Analysis of Variance (ANOVA) showed that population structure explained 39.5% of phenotypic variation (PVE) for tassel main axis length, which was the highest among the 17 agronomic traits included in best linear unbiased prediction (Table S2), indicating vulnerability of this particular trait to the population structure and variable sensitivity of different traits to population structure. Furthermore, heritability (*h^2^*) was highest (0.683) for tassel main axis length (TMAL), while the lowest heritability (0.386) was observed for kernel number per row (KNPR) among the traits. Pair-wise Pearson's correlation coefficients of the 17 traits revealed that phenotypes within a category were more correlated. The values ranged from 0.001 between kernel width and plant height to 0.95 between days to anthesis and heading (Figure S18). These results indicated that all the tested lines possessed significant genetic variability and can be used for further genetic analyses.

### SNP projection and imputation

The whole panel, 513 maize inbred lines, was genotyped using the MaizeSNP50 BeadChip containing 56,110 SNPs (Illumina). RNA sequencing was performed on immature seeds for 368 out of the 513 maize inbreds using 90-bp paired-end Illumina sequencing, resulting in 2,445.9 Gb of raw sequencing data. 556,809 high quality SNPs obtained by combining the two genotyping platforms (RNA-seq and SNP array) [5], [16] were used in the study. For the additional 145 maize lines, the genotype calls of unique loci from the integrated SNP data were projected based on regions of IBD to physical maps constructed using 56110 SNPs, and then high-density markers with more than 0.5 million SNPs were obtained for all the lines. Out of 56,110 SNPs from MaizeSNP50 data set, 49728 SNPs overlapped with the integrated SNPs data based on their physical positions (B73 RefGen_v2). The 49,728 common SNPs were regarded as core or frame markers for projection based on IBD regions. In order to evaluate the performance of IBD [17] based projection, training and validation datasets were established for chromosome 1, which had 7818 core markers from Illumina Maize SNP50 and 88581 SNPs from the integrated data set. The genotypes for one maize line with RNA-seq data in IBD regions were assigned to the matched target line without RNA-seq data for each SNP. The projection accuracy was calculated by comparing inferred genotypes of 368 lines with their real genotype obtained from RNA-seq. In addition, KNN algorithm [4] which infers a large number of missing genotypes generated from low-coverage genome sequencing was used to impute the missing genotypes of the unique loci from RNA-seq SNP data based on 49728 frame markers. Single method analysis, either IBD based projection or KNN algorithm, cannot achieve both optimal accuracy and coverage (see Materials and Methods). However, the combination of IBD based projection and KNN seemed effective to infer a large number of missing genotypes. In order to optimize the set of imputation parameters, a simulation was performed on chromosome 1 in 368 lines (Figure S19). The simulation result on chromosome 1 in 368 lines indicated that the missing rate was reduced from 91.6% (1–7,818/88,581) to 12.8%, with an accuracy rate 96.6% (Table S3). The optimal parameter combination (IBD: SNPs number≥150 in 5 Mb window size; KNN: w = 20, k = 6, p = −7, r = 1) was then used to impute the missing SNPs for the remaining 145 inbred lines, resulting in an 85.5% filling rate. Therefore, our approach combining SNP-chip data and RNA-seq SNP data with an effective projection procedure permits the quick construction of a high-density physical map and integration of SNPs from RNA-seq data set onto the whole population. This approach is also applicable to other genomes and genotyping data from different platforms for a variety of downstream analyses.

### Statistical power of the imputation-based association test

The 368 maize inbreds with 556,809 SNPs genotyped by RNA-seq and Maize SNP50 array were defined as Data set 1. In addition, Data set 1 and 145 maize inbred lines with joint IBD-based projection and KNN imputed genotypes were defined as Data set 2 together. The 513 maize inbreds with Maize SNP50 array genotyped were defined as Data set 3. To evaluate the reliability of imputed genotypes for 145 inbred lines, GWAS was performed using MLM, with both Data sets 1, 2 and 3 focusing on kernel oil concentration, which has been thoroughly analyzed in our previous study [5].

**Figure 1:**
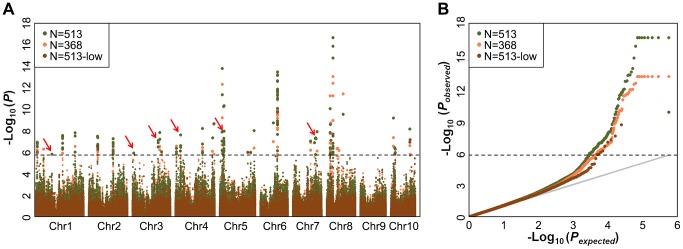
Comparison of mapping results for kernel oil concentration in two different data sets. (A) Manhattan plots of mixed linear model conducted in data set 1 and 2, respectively (data set 1: n = 368, without imputed genotypic data; data set 2: n = 513, 145 lines with imputed genotypic data). The arrow and red boxes indicate the new loci that were not identified in previous study (Li et al, 2013); (B) Quantile-Quantile plots of p-values of mixed linear model conducted in data sets 1 and 2, respectively.

For GWAS performed using MLM with both Data sets 1 to 3, a total of 26, 32 and 8 significant loci were identified in Data sets 1 to 3, respectively, at the Bonferroni-corrected threshold (−log P>5.74, α = 1,) (Figure 1). Almost all strong signals identified in data sets 1 and 3 were also identified in data set 2 (Table S4). More interestingly, we identified six additional significantly associated loci in dataset 2 (−log P>5.74, α = 1), including the phosphoinositide 3-kinases gene (*PI3Ks*) and the phosphatidylinositol transfer protein, which is known to be involved in the oil concentration trait [18] (Figure 1, Table 1, Table S4). This suggests that GWAS carried out using the imputed genotypes with a larger population (n = 513) increased the statistical power compared to the analyses of RNA-seq genotyped SNPs with the smaller population size (n = 368) or low density as DNA array SNPs with the same population size (n = 513).

**Table 1:** Additional SNPs and candidate genes significantly associated with oil concentration found using imputed genotype data.

Candidate gene	Chr	Position	SNP	*Alleles	MAF	eQTL	P(n = 368)	P(n = 513)	Annotation
GRMZM2G132898	1	105672248	M1c105672248	G/T	0.07	N.S.	9.21E-05	1.58E-06	choline-phosphate cytidylyltransferase,CCT
GRMZM2G355572	3	7938035	M3c7938035	A/C	0.09	N.S.	5.39E-06	1.25E-06	Unknown
GRMZM2G142315	3	156963535	M3c156963535	C/G	0.05	6.70E-12	5.42E-06	8.46E-08	phosphatidylinositol transfer protein,CSR1
GRMZM2G122277	4	31245730	PZE-104026172	A/C	0.11	N.S.	1.91E-04′	7.87E-07	Unknown
GRMZM2G138245	5	22806147	M5c22806147	A/G	0.06	8.59E-13	4.77E-05	2.14E-07	Phosphoinositide 3-kinase,PI3Ks
GRMZM2G467356	7	139139746	M7c139139746	C/G	0.06	N.S.	2.93E-05	4.75E-08	Unknown

*Alleles with underlines indicate rare alleles.

N.S: non-significant.

### GWAS for 17 agronomic traits using MLM

GWAS for 17 agronomic traits using MLM was conducted with Data set 2 and the results are summarized in Figures S1C, D, S2, S3, S4, S5, S6, S7, S8, S9, S10, S11, S12, S13, S14, S15, S16, S17C, D. A total of 19 significant SNPs from 10 loci were identified for five traits (ear leaf width, ear length, kernel width, plant height and tassel main axis length) (Table 2). No significant SNP was found to be associated with the other 12 tested traits at the Bonferroni-corrected threshold (−log P>5.74, α = 1). If we set the Bonferrroni-corrected threshold as −log P>7.05 (α = 0.05), no SNPs were significant for all the 17 traits. It may be too strict to use 0.05/n as the cutoff since not all the markers are independent. One thousand permutation tests were conducted for three typical traits with different level of population structure (kernel width, ear height and days to heading) (Table S2). The results showed the cutoff value at α = 0.05 is quite similar (Table S5) with 0.05/n.

**Table 2:** Comparison of significant SNPs identified for 17 traits from MLM using imputed data (dataset 2).

Traits	Chr.	Position	SNPs	Candidate genes	Annotation	−Log_10_ (*P*)*	PVE (%)
EL	1	50646115	A/G	GRMZM2G329040	Haloacid dehalogenase-like hydrolase	6.91	5.32
	1	50668188	A/C	GRMZM2G703565	Thioredoxin-like fold	6.82	5.41
	1	50676605	A/C	GRMZM2G008490	Unknown	6.82	5.2
	1	50712263	T/C	AC208571.4_FG001/GRMZM5G851485	Six-bladed beta-propeller, TolB-like/Helix-loop-helix domain	6.71	3.5
TMAL	1	229858329	T/C	GRMZM2G079428	Unknown	5.76	5.52
	1	285931176	A/G	GRMZM2G434533	Protein kinase, catalytic domain	6.18	5.17
PH	3	162709488	A/G	GRMZM2G401050	Unknown	6.56	9.61
KW	7	148464475	A/C	GRMZM2G413044	Unknown	5.83	0.03
EL	8	101507590	A/G	GRMZM2G164090	Gibberellin regulated protein	6.05	4.23
ELW	10	126586283	A/G	GRMZM2G167280	Protein kinase, catalytic domain	5.81	5.73

*The Bonferroni –corrected threshold is −log_10_ (*P*)>5.74 (the Bonferroni-corrected thresholds for the *P* values were 1.796×10^−6^ and corresponding −log_10_ (*P*) values of 5.74 for 556809 SNPs, at α = 1).

Chr, chromosome; PVE, explained phenotypic variation; EL, ear length; DTH, days to heading; TMAL, tassel main axis length; PH, plant height; ED, ear diameter; KW, kernel width; DTA, days to anthesis; ELW, ear leaf width.

### Anderson-Darling (A-D) test, an alternative method for GWAS

**Figure 2:**
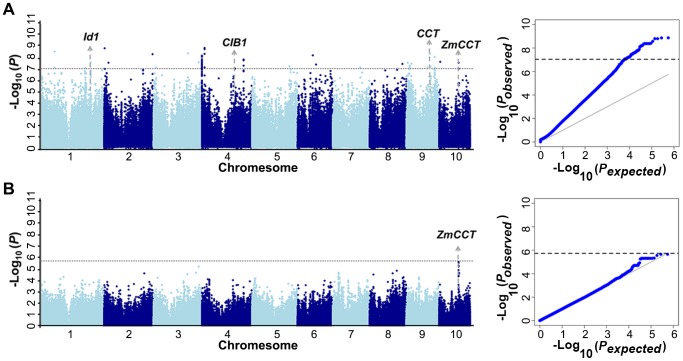
GWAS of the phenotype of days to heading in Yunnan 2010. (A) GWAS result by Anderson–Darling test; (B) GWAS result by mixed linear model.

The A-D test [19] is a nonparametric statistical method and a modification of the KS test [12], [20] that gives more weight to the tails of the distribution than the KS test. Since the identified loci were much less numerous than expected using the MLM method, the data set was reanalyzed using the A-D test. The same three traits (kernel width, ear height and days to heading) were used to perform 1000 time permutation tests to determine the cutoff values. The results showed the cut off value at α = 0.05 varied around the Bonferrroni-corrected threshold as 0.05/n (Table S5). To simplify the procedures, we used the uniform cutoff (−log P>7.05, α = 0.05) for further analysis. Flowering time is an important and well-studied trait, and many QTL or candidate genes have been identified [21], [22]. Recently, several studies have confirmed that *ZmCCT* is the gene underlying the major QTL affecting flowering time on chromosome 10 [22], [23]. Taking flowering time as an example, it provides a good opportunity to test whether A-D test is a feasible GWAS method for agronomic traits or not. Using the A-D test, we identified 30 loci associated with days to heading in Yunnan 2010. Around 20% of 30 loci were located within a QTL support interval reported in NAM population [22]. If the significant loci are randomly distributed in the genome, the probability by chance is equal to the ratio between the whole-length of QTL interval and the whole genome length (12%), which represents an almost twofold enrichment compared with the 12% expected by chance. A strong association (−log_10_ (*P*) = 7.59) was identified in 1.7 Kb upstream of *ZmCCT* (Figure 2A). Four other loci seem to be strong candidates including: one homologous gene (*CIB1*) [24] shown to be involved in the regulation of flowering time in *Arabidopsis*, two homologues containing CCT domain that was demonstrated as key photoperiod regulatory gene in plants [25], and one locus previously shown to affect flowering time in maize (*Id1*) [26] (Figure 2A). Using the MLM method, we were only able to identify the marginally significant association for *ZmCCT* (−log_10_ (*P*) = 5.64) and there were no strong signals in other genome regions (Figure 2B). Therefore, A-D test could be a more appropriate GWAS method for agronomic traits and we performed GWAS using the A-D test in each subpopulation of Data set 2 without controlling of population structure for all tested traits. The total number of unique SNPs significantly associated with the 17 traits was 678, of which 310 represented unique loci (Figure S1E, S2, S3, S4, S5, S6, S7, S8, S9, S10, S11, S12, S13, S14, S15, S16, S17E). The numbers of significant SNPs associated with different traits ranged from 1 (Cob Weight) to 35 (Tassel branch number and Days to silking). For plant height, a total of 107 loci were identified at the Bonferroni-corrected threshold (−log P>7.05, α = 0.05) (Table S6, S7). About 10% of the loci were detected to affect two or more different traits that were consistent with the observed correlations among the measured traits (Figure S18). There were 101, 71 and 171 loci detected in three subpopulations: SS (subpop-1), NSS (subpop-2) and TST (subpop-3), respectively. A reasonable number of spurious associations should be existed in the detected loci since the population structure is not properly addressed in each subpopulation. Genomic control [27]–[30] is a popular method to control the population stratification and cryptic relatedness that was applied to adjust A-D test statistic in each subpopulation in present study. In total, 19 loci were significantly associated with 13 traits at the Bonferroni-corrected threshold (−log P>5.74, α = 1) (Table S7).

**Figure 3:**
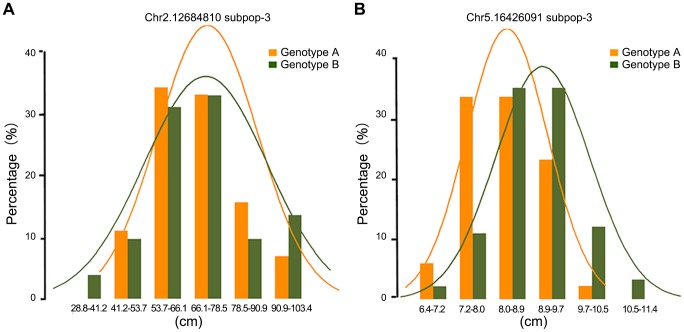
The nature of statistically significant associations. The illustration of associations those are highly significant by Anderson–Darling test, with nearly identical trait means for ear height (A) or with an obvious shift of the means for ear leaf width (B).

To further examine the nature of statistically significant associations, we examined the phenotype distributions of individuals carrying each allele. Interestingly, some associations that differed in the width of the phenotype distribution but which had nearly identical trait means were found to be highly significant by the A-D test but not significant by MLM. Figure 3 illustrated significant loci for ear height with nearly identical trait means (Figure 3A) and significant loci for ear leaf width with an obvious shift of the means (Figure 3B). In total, 14.6% of significant loci identified by A-D test do not have an obvious shift of the mean between the two alleles (t-test, *p*>0.05) (Table S7). In this case the differences between distributions are real, but the corresponding genetic markers would not be useful in breeding if the objective is to change the phenotypic means.

### Comparison of different association mapping methods based on simulated data

Causal allele frequencies and trait distributions are the main factors that affect association mapping efficiency [1], [31]. GWAS data were simulated by adding phenotypic effects to real genotypic data considering the population structure and epistasis from MaizeSNP50 BeadChip [13] under three scenarios: a normal distribution model, an abnormal distribution model caused by uncertain effectors like phenotyping errors and an abnormal distribution model caused by a larger effect QTN with rare alleles (Methods). We compared our noticed A-D test with three other mapping methods: Kruskal-Wallis (K-W) test and linear model (LM) which does not correct for population structure; MLM which corrects for population structure and kinship. Statistic power of the four methods were compared under the same level Type I error. For each method, QTNs were considered to be detected if their *P* value were below the threshold determined by 1,000 times permutation.

**Figure 4:**
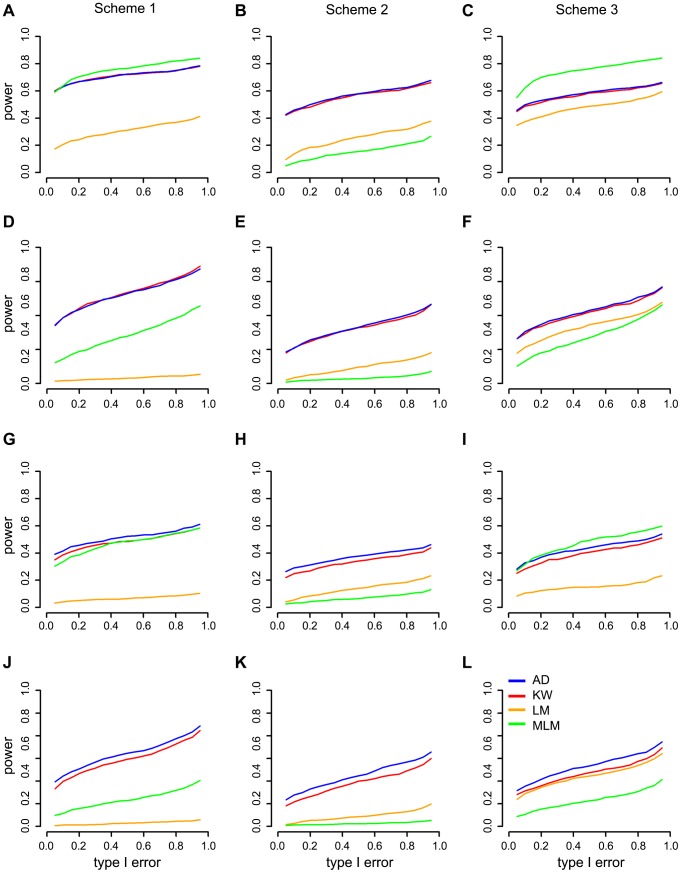
Power comparisons in three simulation schemes for four different mapping methods: A-D, KW, MLM and LM. The “Power” was defined as the detection frequency in 500 repeats for a certain QTN. For the purpose of computing power, a causal SNP was considered to be detected only when the causal SNP was significant at a threshold from 1,000 times permutations. The power and type I error of major QTNs (**A–C**) and moderate QTNs (**D–F**) with common allele frequency. The power and type I error of major QTNs (**G–I**) **a**nd moderate QTNs (**J–L**) with rare allele frequency. A-D test: Anderson–Darling test; LM: linear model; K-W test: Kruskal-Wallis test; MLM: mixed linear model. Scheme 1, phenotypes with normal distribution; Scheme 2, phenotypes with abnormal distribution caused by uncertain effectors; Scheme 3, phenotypes with abnormal distribution caused by a larger effect QTN with rare allele frequency.

The results for the three simulation schemes are shown in Figure 4 and can be summarized as follows: First, MLM has more (Figure 4A, C, I) or similar (Figure 4G) power among the four methods for major QTNs in schemes 1 and 3. Second, regardless of the allele frequency, nonparametric methods usually have greater power than LM and MLM for moderate QTNs (Figure 4D–F, J–I). Third, A-D test is more powerful than K-W test in terms of QTNs with rare alleles (Figure 4J–I). Fourth, nonparametric methods are much more powerful than parametric methods in scheme 2 (Figure 4B, E, H, K) that the phenotype has an abnormal distribution model caused by uncertain effectors.

### Co-localization of QTL and candidate genes for agronomic traits

**Figure 5:**
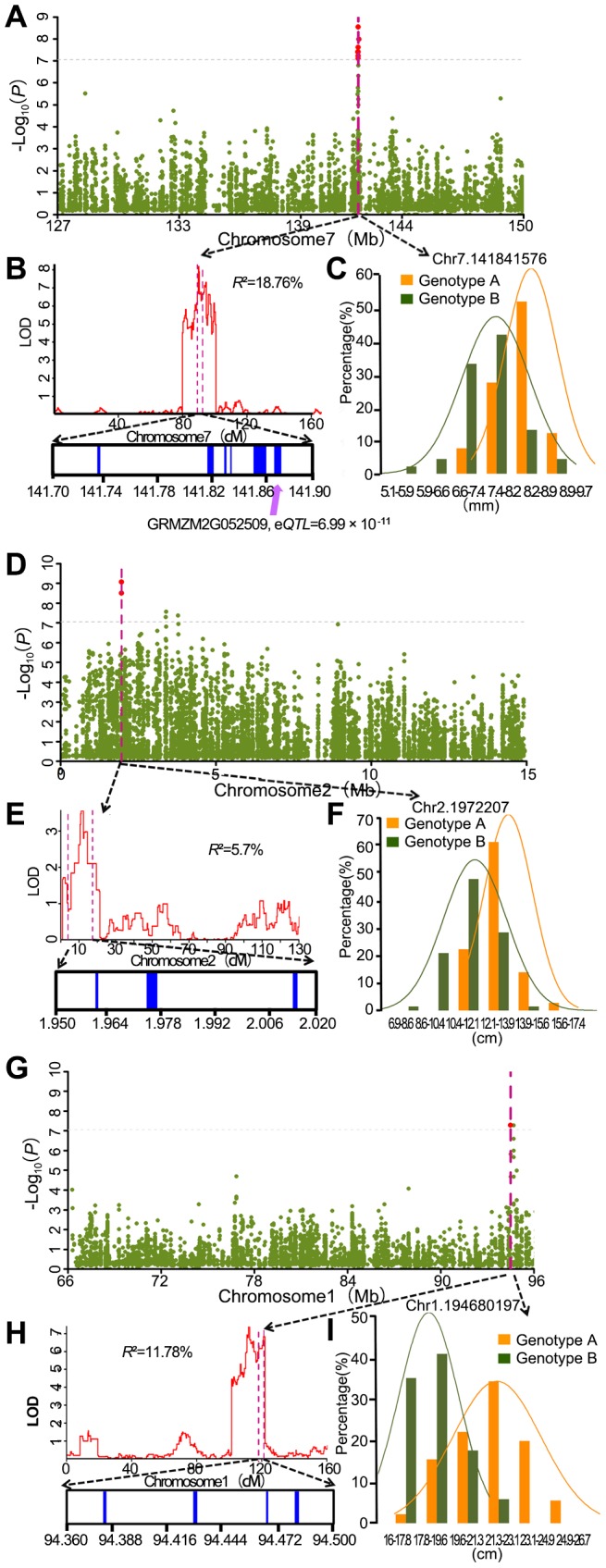
Co-localization of association peaks, QTL and well-annotated candidate genes. **A.** Significant association signals on chromosome 7 for kernel width; **B.** A major kernel width QTL (R^2^ = 18.7%) was mapped on chromosome 7 from 129 Mb to 149 Mb with BK/Yu8701 RILs and covered the significant association signals; **C.** The phenotype's frequency distribution histogram and normal distribution curve at the peak SNP of kernel width; **D.** Significant association signals on chromosome 2 for ear length; **E.** A major ear length QTL (R^2^ = 5.7%) was mapped on chromosome 2 in B73/By804 RILs and covered the significant association signals; **F.** The phenotype's frequency distribution histogram and normal distribution curve at the peak SNP of ear length; **G.** Significant association signals on chr1 for kernel number per row; **H.** A major kernel number per row QTL (R^2^ = 11.78%) was mapped on chr1 in K22/DAN340 RILs and covered the significant association signals; **I.** The frequency distribution histogram and normal distribution curve at the peak SNP of kernel number per row.

We compared our mapping results for 17 agronomic traits with QTL identified using different linkage segregation populations and with previously reported known genes. Loci identified by the A-D test which overlap with previously identified genes and loci mapped in biparental populations are summarized in Table S7. Considering the large confidence interval of previously reported QTL, 3 independent RIL populations genotyped with high-density SNP markers were used to conduct QTL analysis for 3 traits (kernel width, ear length and kernel number per row) of the 17 traits tested. For the compared traits, 9 loci (20% of the detected loci) identified by the A-D test were within the QTL confidence interval. One example is a major kernel width QTL which was mapped in chromosome 7 with BK/Yu8701 RILs and explains 18.7% of phenotypic variation (Figure 5B). Within the QTL interval, significant SNPs- kernel width association was detected (Figure 5A). Six candidate genes: GRMZM2G354539, GRMZM2G052893, GRMZM2G052817, GRMZM2G354525, GRMZM2G052610, and GRMZM2G052509 are found in the associated interval. An expression quantitative trait locus (eQTL) was detected for one (GRMZM2G052509, −log_10_ (*P*) = 10.16) of the six annotated genes, which can therefore be regarded as a candidate gene for further study. The second example of overlap included the SNP chr2.s_1972207(C/G) with −log_10_ (*P*) = 9.06and SNP chr2.s_1972176(C/G) with −log_10_ (*P*) = 8.49 which were both significantly associated with ear length, and a QTL affiliated with ear length identified in B73/By804 RIL near the associated peak (Figure 5D–F). SNP chr2.s_1972207 (C/G) and SNP chr2.s_1972176 (C/G) were the only two of the 36 SNPs within the gene GRMZM2G061877, which encodes a DHHC zinc finger domain containing protein, and both of them are in the CDS region. SNP chr2.s_1972176 (C/G) makes no difference to the translated protein sequence, while SNP chr2.s_1972207 (C/G) results in a change of Isoleucine to Methionine. Several zinc finger proteins that play important roles in maize inflorescence development, for instance transcription factors *RA1*, *RA2* and *RA3* in ramosa pathways [32], have been identified. In rice, a zinc finger transcription factor *DST* directly regulates *OsCKX2* expression in the reproductive meristem leading to *OsCKX2* regulated CK accumulation in the shoot apical meristem (SAM) and, therefore, controls the number of the reproductive organs; the *dst* mutant leads to lower plant height and longer rice panicle length [33]. These zinc finger genes are functioning as transcription factors. Since the DHHC protein domain product of GRMZM2G068177, which was strongly suggested as a candidate gene for the regulation of ear length, acts as an enzyme, this may suggest a novel function of zinc finger proteins in monocot reproductive organ development. However, further work is needed to test this hypothesis. The third example, one QTL located on chromosome 1 using K22/Dan340 RIL population which explains 11.8% of phenotypic variation for kernel number per row, also overlapped with significant association signals (Figure 5G–I) Four candidate genes: GRMZM2G088524, GRMZM2G022822, GRMZM2G108180 and GRMZM2G052666, located in a 200 kb window around the significant signals, were predicted.

## Discussion

The genome-wide imputation of genotypes has attracted much attention given its broad applicability in the GWAS era. There are a number of methods for imputing missing genotypes, but many factors influence the accuracy of imputed genotypes [34], [35]. In this study, we proposed a two-step method combining IBD based projection and KNN algorithm to infer missing genotypes, resulting in 96.6% accuracy and 85.5% genome coverage in the tested samples. Considering that the missing genotypes consist of over 91.6% of our raw data set, this level of accuracy is acceptable. Compared with other methods [4], [17], [34], [35], the two-step method has its advantages. Imputation based only on IBD regions ensures high accuracy but a relatively low coverage rate. KNN algorithm has been proven to be a good strategy for sequencing data [4], however, it alone does not represent the true similarity of the inbred lines due to low density of frame markers and rapid LD decay in maize in our study. Therefore, we first used IBD based imputation to increase marker density, and then the KNN algorithm was used to infer the missing genotype, leading to high coverage rate and imputation accuracy. Imputation error is often caused by ignoring recombination and mutation within IBD regions. In addition, if an inbred line with low density markers share a region with two or more inbred lines with high density markers and the missing genotypes are inferred on the basis of only one of these lines, there is a high risk of error since the accuracy of the projection depends on the identity between the projected and chosen lines. Reanalysis of GWAS for kernel oil concentration revealed consistent results and a higher detection power. Six more associated loci were identified, most likely due to the increase in sample size. The implication is that mapping resolutions are enhanced by extracting moderately more information from the genome and expanding sample size.

The detection of loci controlling complex traits using GWAS has flourished and numerous statistical approaches for GWAS analysis in plants have recently been described [14], [36]–[40]. Linear statistical models like ANOVA, general linear model (GLM), and MLM establish significance cutoff by relying on the assumption that target traits have normal distribution. However, sometimes phenotype distribution in the moderate plant population is not normal in the tails that may be due to the population size, field experiment such as phenotyping errors, or genetic effects [31]. Based on our simulated data, nonparametric methods including A-D and KW tests usually have greater power than LM and MLM for abnormal phenotypes, rare alleles and moderate QTNs. It also implies that A-D and KW tests should perform well to detect the shifts of distribution as well as changes in the shape of distributions [31]. A-D test possesses advantages than K-W test in the detection of QTNs with rare alleles. However, MLM performs better than A-D and KW test for the major QTNs especially those with common alleles. However, we need to keep in mind that population structure of the studied samples is the key confounder for GWAS. In the measured 17 agronomic traits of present study, we observed the phenotypic variation explained by population structure ranged between 0.9% and 32.3% (Table S2). In the A-D test, we didn't account the confounding by population structure in the subpopulation that may lead to false-positive findings. Genomic control is a good alternative for controlling the statistic inflations [27]–[30], different inflation factors were observed in different traits and different subpopulations in present study (Table S7). We detected 19 loci significantly associated with 13 traits at the Bonferroni-corrected threshold (−log P>5.74, α = 1) (Table S7) using genomic-control (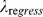) to adjust our real phenotype test statistic from A-D test. However, we also need to be careful that the adjusted −Log P might be over corrected, since A-D test has already controlled part of the population structure and genomic control method is affected significantly by the true association signals , even for the agronomic traits may involve a larger number of loci with small effects [21], [36], [37], [41]. And the influence of epistatic genetic effect to the genomic control is still not explored [27]–[30]. Another thing need to be noticed is testing within subpopulations (A-D test) and across the whole panel with controlling the population structure (MLM) are different. Testing within subpopulations changes allele frequencies of background alleles and therefore possibly changes the epistatic interactions that are mapped in an additive manner within subpopulations but were not mapped across populations.

In general, A-D test could be a good complement to current popular GWAS methods. As each method owning its own advantages, the preliminary understanding of the traits studied is needed for choosing GWAS methods or trying different GWAS methods would be helpful especially for those studies only few or none significant signals were identified by using only one method. In this study, we performed GWAS using both MLM and A-D test for 17 agronomic traits. In total, 18 overlapped regions were detected by the two approaches (Table S7). The A-D test also showed high concordance with previous studies in identifying a higher number of QTL related to agronomic traits.

Our noticed nonparametric statistical approach is robust with respect to non-normality, similarly to the KS test [12]. The KS test tends to be more sensitive around the median value and less sensitive at the extreme ends of the distribution. Thus, the KS test is not always appropriate for calculating the significance of data sets which differs at the tails of the probability distribution, while the median remains unchanged [31]. The A-D test improves upon the KS test because it has more sensitivity towards the tails of the pooled sample. More importantly, the performance of the A-D test for small samples is quite good, as demonstrated by numerous Monte Carlo simulations [19]. This means that, for complex traits, the A-D test can make a good use of SNPs that have minor allele frequency and keep detection ability to the relatively small effect loci. At the same time, it is important to recognize that there are always limitations to what can be achieved using statistics. It seems that A-D test does not work well for all traits. Interestingly, we identified 14.6% associations by A-D test that differed in the width of the phenotype distribution but which had nearly identical trait means (Figure 3A). In these cases the differences between distributions are real, but the corresponding genetic markers would not be useful in breeding if the objective is to change the phenotypic means. Instead, the associations appear to represent allelic differences in the apparent trait stability. Therefore, to confirm candidate loci, it is necessary to check both frequency distribution and normality of the distribution curves (Figure 3). Several studies in humans have confirmed that using multiple methods for statistical inference critically enables the interpretation of results and engenders stronger candidates for experimental follow-up [42].

We identified some genes affecting important agronomic traits in maize that are very good candidates for future detailed analysis, for allele mining to identify functional variation, and for marker development. As whole genome sequences become available for many crop species including maize, as well as for multiple genotypes of the same species through resequencing, along with cost-effective high-throughput genotyping systems and the next generation of sequencing technologies, GWAS becomes practical and its use in plant breeding will allow the manipulation of many traits at the whole-genome level. Association mapping using a set of global diverse breeding germplasm and high-throughput SNP markers, as shown in this study, provides high-resolution dissection of the genetic architecture of complex traits. This knowledge in turn will be useful not only for designing marker-assisted selection strategies but also for optimizing conventional breeding systems.

## Materials and Methods

### Plant material and phenotyping

A total of 513 maize lines with tropical, subtropical and temperate backgrounds representing the global maize diversity were employed for genome-wide association mapping in this study. All maize inbred lines have been well described in previous studies [13], [15] and the 513 maize lines were classified into four subgroups based on population structure Q matrix: Stiff stalk (SS) with 112 lines, Non-stiff stalk (NSS) with 116 lines, Tropical-subtropical (TST) with 258 lines, and an admixed group with 27 lines (detailed information also can be downloaded at (www. maizego.org/resource). A Randomized complete block design with one to two replications was used for field trials in five environments, including Ya'an (30°N, 103°E), Sanya (18°N, 109°E), Yunnan (25°N, 102°E) in 2009, Guangxi (23°N, 110°E) and Yunnan (25°N, 102°E) in 2010. A row length of 3 m was used for each line including 11 plants plot^−1^ with 25 cm plant to plant and 60 cm row to row distance. Five randomly selected plants were used for phenotypic data acquisition in each line and the mean data in each replication was used for phenotypic analysis. A total of 17 economically important traits were phenotyped (Table S1). These traits were divided into three categories: morphological attributes (plant height, ear height, ear leaf width and length, tassel main axis length, tassel branch number, and leaf number above ear), yield related traits (ear length and diameter, cob diameter, kernel number per row, 100-grain weight, cob weight and kernel width), and maturity traits (days to heading, anthesis, and silking). Best linear unbiased predictions (BLUP) for each line across five environments were calculated using the MIXED procedure in SAS (Release 9.1.3; SAS Institute, Cary, NC), and employed for evaluating trait variation in the association panel.

### Imputation yield and accuracy

Imputation methods have not been developed to deal specifically with low density of SNP marker data. Of the available imputation models, identity by descent (IBD) based projection [17] and the k-nearest neighbor algorithm (KNN) [4] seemed to effectively infer a large number of missing genotypes. To assess the performance of IBD based projection, preliminary tests for chromosome 1 in 368 maize lines were conducted. We removed genotype data without frame SNPs and then compared the observed genotypes with those generated by projection. The number of IBD regions with consecutive SNPs for 368 lines varied from 1 to 285 on chromosome 1, and projection accuracy, defined as the percentage of correctly projected genotypes ranged from 74.8% to 99.1%, with an average of 92.6% (Table S3). The mean error ratio pooled over in 32,015 IBD regions on chromosome 1 for the 368 maize lines was also calculated (Figure S19A, B), and gradually declined with the increasing number of identical SNP and size in IBD regions. Preliminary testing suggested that IBD regions with 150 consecutive SNPs and a size of 5 Mb or more were highly conserved in maize and error rate for projection was well controlled within 5% (Figure S19A, B). The number of qualified IBD segments ranged from 0 to 20 for 368 lines and coverage rate, defined as the percentage of projected genotypes, accounted for 61.99% of genomic regions on chromosome 1, with projection accuracy increased from 92.59% to 96.62% on average (Table S3). Therefore, IBD based projection for regions with 150 consecutive SNPs and a size of 5 Mb were applied for integration of SNPs from RNA-seq data set onto the 145 additional maize inbred lines. Alternatively, the K-Nearest Neighbor (KNN) algorithm was also used to enrich the physical map of each line constructed by 56,110 SNPs from MaizeSNP50 chip by inferring the missing genotypes of the unique loci from RNA-seq SNP data. In the preliminary test, this method was efficient and the imputation accuracy and coverage rate for 368 lines were 97.48% and 75.35%, respectively (Table S3). The IBD based projection and KNN imputation revealed high inferred accuracy; however, the coverage rates were relatively low, with an average of 62% and 75%, respectively. In order to increase the coverage rate and keep high imputation accuracy, IBD based projection and KNN algorithm were combined to infer missing genotypes. The IBD method can provide more frame SNPs for the KNN algorithm, and simultaneously the KNN algorithm compensates for the weakness of the IBD method in coverage rate. About 38% of the genotypes were missing after prediction of IBD regions with 150 consecutive SNPs and 5 Mb size, and then the KNN algorithm was used to impute the missing data, resulting in 95.8% of accuracy for the missing data. The joint IBD based projection and KNN imputation of the genotypes of 368 lines increased coverage rate from 62% to 87.2%, with a total accuracy of 95.9% in the preliminary test. The projection accuracy was also affected by heterozygosity of each line, which increased from 95.88% to 96.60% after excluding 44 lines with more than 10% heterozygosity. The joint IBD based projection and KNN imputation that performed well in the preliminary test was used for the integration of SNPs from the high density SNPs data set onto 145 maize lines genotyped by 56110 SNPs. For 145 maize lines, 54.18% and 32.28% of loci across 10 chromosomes were inferred through IBD based projection and subsequent KNN imputation, respectively. As a result, 85.46% of loci for the whole maize genome were filled. The average density for the whole panel increased from 20 SNPs to more than 200 SNPs per Mb.

### QTL mapping

The linkage analyses of ear length, kernel number per row, and kernel width were performed in three recombination inbred line (RIL) populations, BY804/B73 (197 individuals), K22/Dan340 (197 individuals), and BK/Yu8701 (165 individuals). All the RIL lines and their parents were genotyped using Maize SNP50 assays (Illumina) containing 56,110 SNPs [14]. The phenotype of BK/Yu8701 in Henan 2011 and BLUP value from 5 environments of BY804/B73 and K22/Dan340 were used. QTL mapping using the composite interval mapping method [43] was performed in the package QTL cartographer version 2.5 [44].

### Statistical analysis and association mapping

ANOVA, correlation, and repeatability analyses for 17 agronomic traits were conducted using SAS software (Release 9.1.3; SAS Institute, Cary, NC). Heritability analysis and association analysis for the 17 agronomic traits in Data set 2 were conducted by MLM using TASSEL [45] software package. The observed *p* values from marker-trait associations were used to display Q-Q plots and Manhattan plots, using R. Permutation tests were used to determine the cutoff for GWAS. Considering the computation time, we only choose three typical traits with different population structure effects (kernel width, ear height and day of flowering time) as examples. The results showed that the cutoff values are similar with the Bonferroni correction. To simplify the procedures, we use the uniform Bonferroni-corrected thresholds at α = 1 and α = 0.05 as the cutoffs. When performing n tests, if the significance level for the entire series of tests is α, then each of the tests should have a probability of P = α/n. When the numbers of markers was 556809 SNPs, at α = 1 and α = 0.05, the Bonferroni-corrected thresholds for the *p* values were 1.796×10^−6^ and 8.95×10^−8^, with corresponding −log *p* values of 5.74 and 7.05, respectively. Regression estimator (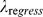) of Genomic Control inflation factor was used [28]. Percentage of PVE by associated SNPs was calculated by ANOVA. Informative SNPs and candidate genes at the identified loci for the corresponding traits were from public maize genome data set B73 RefGen_v2.

### Simulation study

To compare the power and FDR of A-D test, Kruskal-Wallis (K-W test) test, linear model (LM) and mixed linear model (MLM), three schemes with different phenotype distribution were simulated by considering the QTN effects and allele frequency.

Scheme 1 was used to simulate a normal distribution phenotype with the contribution of population structure, additive genetic effect, epistatic genetic effect and residual effect [6]. The population structure and epistasis explained 10% of the total phenotypic variation, respectively. The additive effect was the sum of all additive effects for 20 causal QTNs. For approaching the real genetic architecture, we set 20% major QTNs explaining 30% of the sum of all assigned genetic effect and 80% moderate QTNs explaining 70% of the sum of all assigned genetic effect. Half of major and moderate QTNs were rare alleles (MAF = 0.05–0.1) and half were common alleles (MAF = 0.25–0.45). Larger genetic effects were assigned to the rare alleles QTNs to ensure them could explain the same proportion of phenotypic variation as common alleles QTNs. The ratio of assigned genetic effects between rare alleles QTNs (at MAF = 0.075) and common alleles QTNs (at MAF = 0.35) was calculated based on 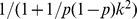. The genetic effect was assigned to all SNPs, one at a time [6]. The proportion of the additive effect was defined by narrow-sense heritability which is the proportion of additive variance over the total variance, and 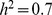 was examined. The residual effect followed a normal distribution and had a variance to satisfy the contributions from additive and epistatic effects at the designated level [6].

Scheme 2 was used to simulate an abnormal distribution phenotype with a long tail on one side. On the basis of scheme 1, 10% of lines were randomly selected and added an extra residual effect (1 to 6 fold standard deviation of the phenotype). All the others were same.

Scheme 3 was designed to simulate an abnormal distribution phenotype caused by a larger effect background rare QTN. The additive effect was still the sum of all additive effects for 20 causal QTNs. 1 background QTN, 3 major QTNs and 16 moderate QTNs explaining 25%, 20%, 60% of the sum of all assigned genetic effect respectively. The population structure effect, epistatic effect and residual effect were consistent with scheme 1.

Simulations of the phenotypes were repeated 500 times in all schemes. All simulated phenotypes had been analyzed with the four methods presented in the main text. 1,000 permutations had been done separately for the four methods to obtain the threshold at different type I error risk.

### Anderson-Darling test

The Anderson-Darling two-sample procedure assumes that the two samples have a continuous distribution function and we are interested in testing the null hypothesis that the two phenotype samples divided by two alleles of one SNP have the same distribution, without specifying the nature of population: 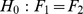


The test procedure is as follows:

1. Calculate 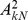:

The computational formula for 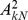 not adjusted for ties is,
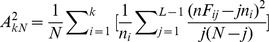
and the corresponding adjusted for ties is,
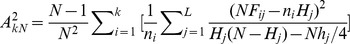
where:


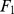
, 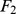 indicates the two phenotype distribution function

k = 2; i = 1, 2


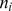
 = data number in the *i*th sample; j = 1,2,…,
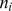


N = total number of two samples' individuals; 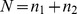



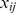
 = data in the i sample and j observation within that sample

L = the number of unique data, where it will be less than n with tied data

z(j) = distinct values of all combined data ordered in ascendant way denoted z(1),z(2),…,z(L)


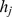
 = number of values in the pooled sample equal to z(j)


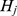
 = number of values in the combined samples less than z(j) plus one half of the number of values in the combined samples equal to z(j)


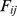
 = number of values in the *i*th sample which are small than z(j) plus one half the number of values in this sample which are equal to z(j)

2. Calculate 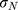:

Under 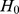, the variance of 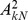 is,
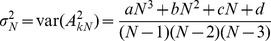
with:
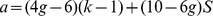


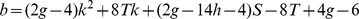


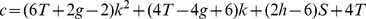


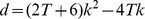
where:
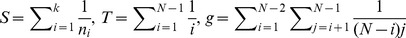



3. Calculate 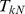:
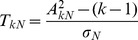



4. Refer 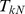 to the upper α percentiles 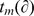 of the 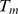 distribution table below, reject 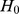 at significance level 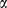 if 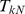 exceeds the given point 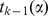.If 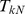 is outside the range of the table. Plotting the log-odds of 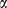 versus 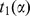, a strong linear pattern indicates that simple linear extrapolation should give good approximate *p* values.
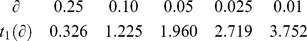
where:
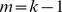



**URL.** *One R package (ADGWAS) for GWAS by Anderson-Darling test can be downloaded here:* http://www.maizego.org/Resources.html

## Supporting Information

Figure S1 Genome-wide association analysis of plant height. (**A, B**) Phenotype histogram and distribution of subpopulations in 513 maize lines. (**C**) Manhattan plots of mixed linear model conducted in imputation data, respectively. (**D**) Quantile-Quantile plots of p-values of mixed linear model conducted in imputation data. Know genes controlling the traits were labeled. (**E**) Summary of GWAS results from Anderson-Darling test performed on each subpopulation independently for plant height. The three subpopulations: SS (subpop-1), NSS (subpop-2) and TST (subpop-3).
(TIF)

Figure S2 Genome-wide association analysis of ear height. (**A, B**) Phenotype histogram and distribution of subpopulations in 513 maize lines. (**C**) Manhattan plots of mixed linear model conducted in imputation data, respectively. (**D**) Quantile-Quantile plots of p-values of mixed linear model conducted in imputation data. (**E**) Summary of GWAS results from Anderson-Darling test performed on each subpopulation independently for ear height.
(TIF)

Figure S3 Genome-wide association analysis of ear leaf width. (**A, B**) Phenotype histogram and distribution of subpopulations in 513 maize lines. (**C**) Manhattan plots of mixed linear model conducted in imputation data, respectively. (**D**) Quantile-Quantile plots of p-values of mixed linear model conducted in imputation data. (**E**) Summary of GWAS results from Anderson-Darling test performed on each subpopulation independently for plant ear leaf width.
(TIF)

Figure S4 Genome-wide association analysis of ear leaf length. (**A, B**) Phenotype histogram and distribution of subpopulations in 513 maize lines. (**C**) Manhattan plots of mixed linear model conducted in imputation data, respectively. (**D**) Quantile-Quantile plots of p-values of mixed linear model conducted in imputation data. (**E**) Summary of GWAS results from Anderson-Darling test performed on each subpopulation independently for ear leaf length.
(TIF)

Figure S5 Genome-wide association analysis of tassel main axis length. (**A, B**) Phenotype histogram and distribution of subpopulations in 513 maize lines. (**C**) Manhattan plots of mixed linear model conducted in imputation data, respectively. (**D**) Quantile-Quantile plots of p-values of mixed linear model conducted in imputation data. Know genes controlling the traits were labeled. (**E**) Summary of GWAS results from Anderson-Darling test performed on each subpopulation independently for tassel main axis length.
(TIF)

Figure S6 Genome-wide association analysis of tassel branch number. (**A, B**) Phenotype histogram and distribution of subpopulations in 513 maize lines. (**C**) Manhattan plots of mixed linear model conducted in imputation data, respectively. (**D**) Quantile-Quantile plots of p-values of mixed linear model conducted in imputation data. Know genes controlling the traits were labeled. (**E**) Summary of GWAS results from Anderson-Darling test performed on each subpopulation independently for tassel branch number.
(TIF)

Figure S7 Genome-wide association analysis of leaf number above ear. (**A, B**) Phenotype histogram and distribution of subpopulations in 513 maize lines. (**C**) Manhattan plots of mixed linear model conducted in imputation data, respectively. (**D**) Quantile-Quantile plots of p-values of mixed linear model conducted in imputation data. (**E**) Summary of GWAS results from Anderson-Darling test performed on each subpopulation independently for leaf number above ear.
(TIF)

Figure S8 Genome-wide association analysis of ear length. (**A, B**) Phenotype histogram and distribution of subpopulations in 513 maize lines. (**C**) Manhattan plots of mixed linear model conducted in imputation data, respectively. (**D**) Quantile-Quantile plots of p-values of mixed linear model conducted in imputation data. Know genes controlling the traits were labeled. (**E**) Summary of GWAS results from Anderson-Darling test performed on each subpopulation independently for ear length.
(TIF)

Figure S9 Genome-wide association analysis of ear diameter. (**A, B**) Phenotype histogram and distribution of subpopulations in 513 maize lines. (**C**) Manhattan plots of mixed linear model conducted in imputation data, respectively. (**D**) Quantile-Quantile plots of p-values of mixed linear model conducted in imputation data. Know genes controlling the traits were labeled. (**E**) Summary of GWAS results from Anderson-Darling test performed on each subpopulation independently for ear diameter.
(TIF)

Figure S10 Genome-wide association analysis of cob diameter. (**A, B**) Phenotype histogram and distribution of subpopulations in 513 maize lines. (**C**) Manhattan plots of mixed linear model conducted in imputation data, respectively. (**D**) Quantile-Quantile plots of p-values of mixed linear model conducted in imputation data. Know genes controlling the traits were labeled. (**E**) Summary of GWAS results from Anderson-Darling test performed on each subpopulation independently for cob diameter.
(TIF)

Figure S11 Genome-wide association analysis of kernel number per row. (**A, B**) Phenotype histogram and distribution of subpopulations in 513 maize lines. (**C**) Manhattan plots of mixed linear model conducted in imputation data, respectively. (**D**) Quantile-Quantile plots of p-values of mixed linear model conducted in imputation data. Know genes controlling the traits were labeled. (**E**) Summary of GWAS results from Anderson-Darling test performed on each subpopulation independently for kernel number per row.
(TIF)

Figure S12 Genome-wide association analysis of 100-grain weight. (**A, B**) Phenotype histogram and distribution of subpopulations in 513 maize lines. (**C**) Manhattan plots of mixed linear model conducted in imputation data, respectively. (**D**) Quantile-Quantile plots of p-values of mixed linear model conducted in imputation data. Know genes controlling the traits were labeled. (**E**) Summary of GWAS results from Anderson-Darling test performed on each subpopulation independently for 100-grain weight.
(TIF)

Figure S13 Genome-wide association analysis of cob weight. (**A, B**) Phenotype histogram and distribution of subpopulations in 513 maize lines. (**C**) Manhattan plots of mixed linear model conducted in imputation data, respectively. (**D**) Quantile-Quantile plots of p-values of mixed linear model conducted in imputation data. Know genes controlling the traits were labeled. (**E**) Summary of GWAS results from Anderson-Darling test performed on each subpopulation independently for cob weight.
(TIF)

Figure S14 Genome-wide association analysis of kernel width. (**A, B**) Phenotype histogram and distribution of subpopulations in 513 maize lines. (**C**) Manhattan plots of mixed linear model conducted in imputation data, respectively. (**D**) Quantile-Quantile plots of p-values of mixed linear model conducted in imputation data. (**E**) Summary of GWAS results from Anderson-Darling test performed on each subpopulation independently for kernel width.
(TIF)

Figure S15 Genome-wide association analysis of days to anthesis. (**A, B**) Phenotype histogram and distribution of subpopulations in 513 maize lines. (**C**) Manhattan plots of mixed linear model conducted in imputation data, respectively. (**D**) Quantile-Quantile plots of p-values of mixed linear model conducted in imputation data. Know genes controlling the traits were labeled. (**E**) Summary of GWAS results from Anderson-Darling test performed on each subpopulation independently for days to anthesis.
(TIF)

Figure S16 Genome-wide association analysis of days to silking. (**A, B**) Phenotype histogram and distribution of subpopulations in 513 maize lines. (**C**) Manhattan plots of mixed linear model conducted in imputation data, respectively. (**D**) Quantile-Quantile plots of p-values of mixed linear model conducted in imputation data. (**E**) Summary of GWAS results from Anderson-Darling test performed on each subpopulation independently for days to silking.
(TIF)

Figure S17 Genome-wide association analysis of days to heading. (**A, B**) Phenotype histogram and distribution of subpopulations in 513 maize lines. (**C**) Manhattan plots of mixed linear model conducted in imputation data, respectively. (**D**) Quantile-Quantile plots of p-values of mixed linear model conducted in imputation data. Know genes controlling the traits were labeled. (**E**) Summary of GWAS results from Anderson-Darling test performed on each subpopulation independently for days to heading.
(TIF)

Figure S18 Pair-wise Pearson's correlation among 17 traits in 513 maize lines.
(TIF)

Figure S19 The mean error ratio and mean coverage ratio pool over SNP number within IBD region (**A**) and size of IBD region (**B**), respectively, on chromosome 1 for the 368 maize lines. (**C**) Imputation accuracy and filling rate for each of 72 combinations of variables of KNN. The combination, indicated by arrow, was chosen for final data imputation.
(TIF)

Table S1 Description of the traits evaluated in the study.
(XLSX)

Table S2 Phenotype variation of 17 agronomic traits in 513 maize lines. ^a^ ANOVA, analysis of variance, showing the mean square and degrees of freedom (in parentheses). The F-test was applied to determine the significance level. Both the environments and lines were fitted in the model as random effects. ** indicate significance at level of 0.001; s.d., standard deviation. ^b^PVE by Q, the percentage of phenotypic variance explained by the subpopulation structure.
(XLSX)

Table S3 IBD base projection and KNN imputation for validation dataset.
(XLSX)

Table S4 The comparison of significantly associated loci with oil concentration in dataset 1 (N = 368) and dataset 2 (N = 513). *Alleles with underlines indicate rare alleles. NS: non-significant. NA: no SNPs.
(XLSX)

Table S5 Cut off value (alpha = 5%) defined by 1000 permutations and detected QTNs associated with three different traits using MLM and A-D test based on 50K SNPs, respectively.
(XLSX)

Table S6 The summary of significant SNP number detected by Anderson-Darling test (A-D test, −log P>7.05) and mixed linear model (MLM, −log P>4) in 513 maize inbreds. *shared SNPs and loci detected by both methods.
(XLSX)

Table S7 Significant loci detected by A-D test. SS: Stiff stalk; NSS: Non-stiff stalk; TST: Tropical-subtropical.
(XLSX)
