# Supplementary figures and images for: Genome Wide Association Studies Using a New Nonparametric Model Reveal the Genetic Architecture of 17 Agronomic Traits in an Enlarged Maize Association Panel
Source: PLoS Genet. 2014 Sep 11;10(9):e1004573. doi: 10.1371/journal.pgen.1004573 (PMC4161304; doi:10.1371/journal.pgen.1004573)

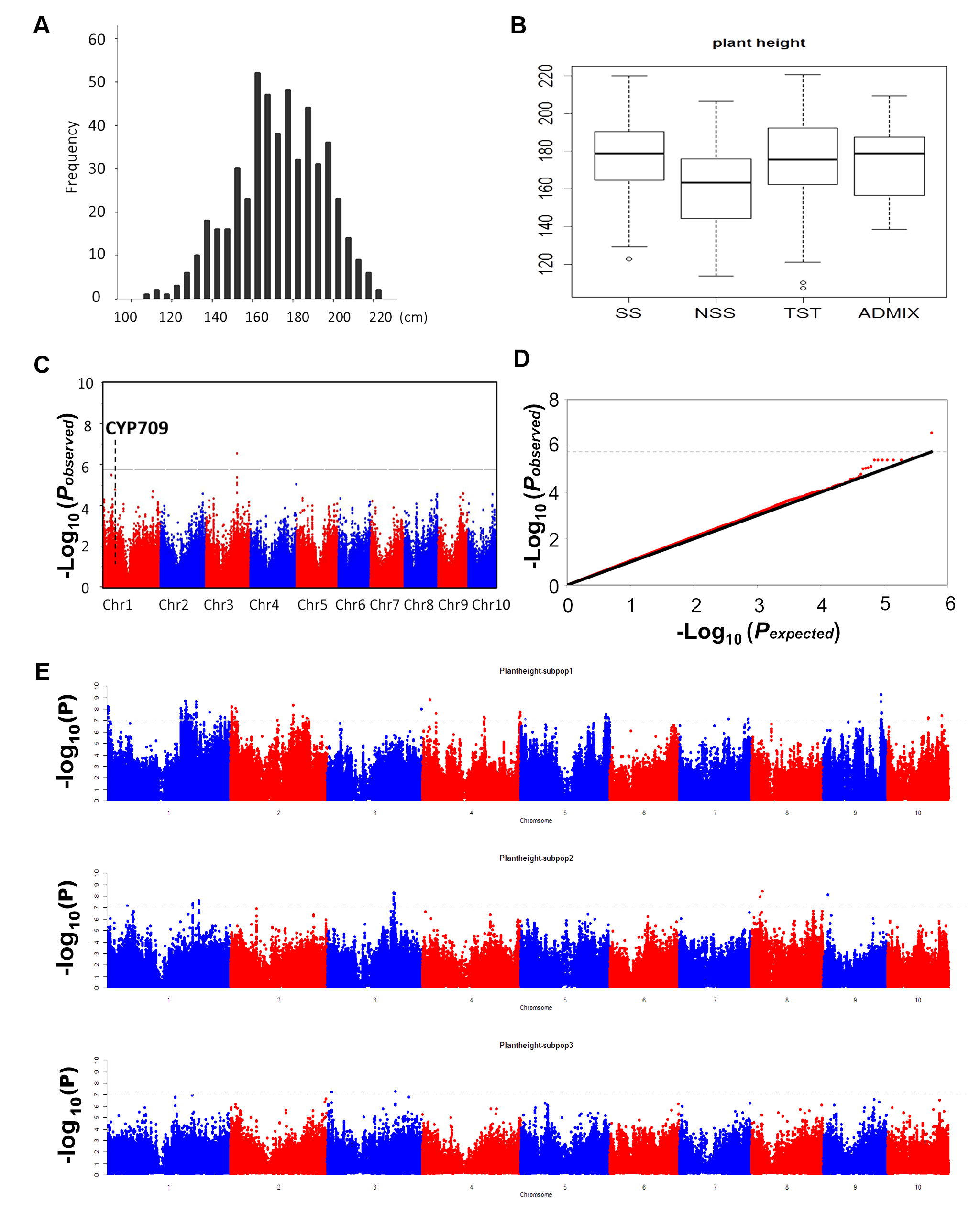

Supplement: Figure S1 — Genome-wide association analysis of plant height. (A, B) Phenotype histogram and distribution of subpopulations in 513 maize lines. (C) Manhattan plots of mixed linear model conducted in imputation data, respectively. (D) Quantile-Quantile plots of p-values of mixed linear model conducted in imputation data. Know genes controlling the traits were labeled. (E) Summary of GWAS results from Anderson-Darling test performed on each subpopulation independently for plant height. The three subpopulations: SS (subpop-1), NSS (subpop-2) and TST (subpop-3). (TIF) [file pgen.1004573.s001.tif]

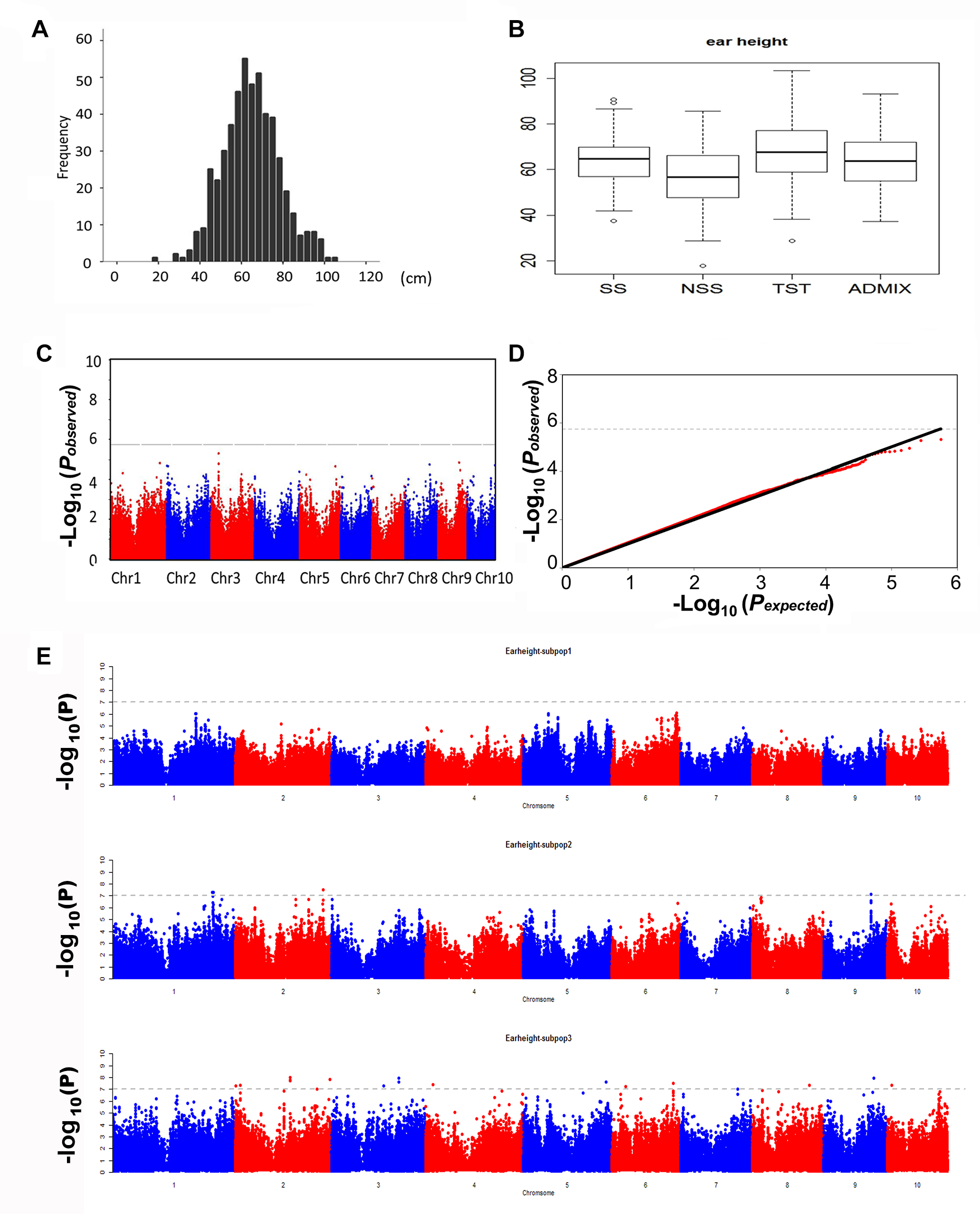

Supplement: Figure S2 — Genome-wide association analysis of ear height. (A, B) Phenotype histogram and distribution of subpopulations in 513 maize lines. (C) Manhattan plots of mixed linear model conducted in imputation data, respectively. (D) Quantile-Quantile plots of p-values of mixed linear model conducted in imputation data. (E) Summary of GWAS results from Anderson-Darling test performed on each subpopulation independently for ear height. (TIF) [file pgen.1004573.s002.tif]

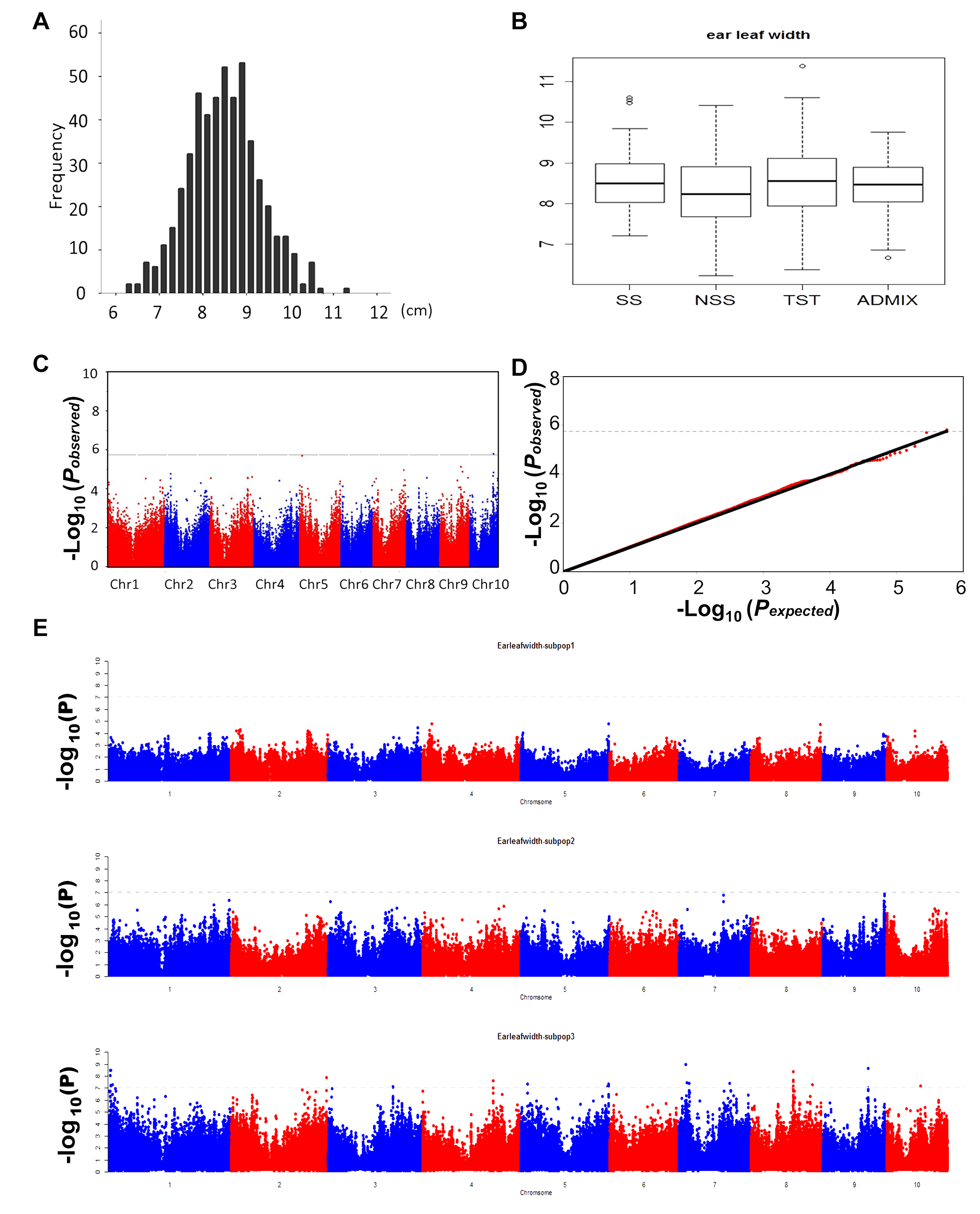

Supplement: Figure S3 — Genome-wide association analysis of ear leaf width. (A, B) Phenotype histogram and distribution of subpopulations in 513 maize lines. (C) Manhattan plots of mixed linear model conducted in imputation data, respectively. (D) Quantile-Quantile plots of p-values of mixed linear model conducted in imputation data. (E) Summary of GWAS results from Anderson-Darling test performed on each subpopulation independently for plant ear leaf width. (TIF) [file pgen.1004573.s003.tif]

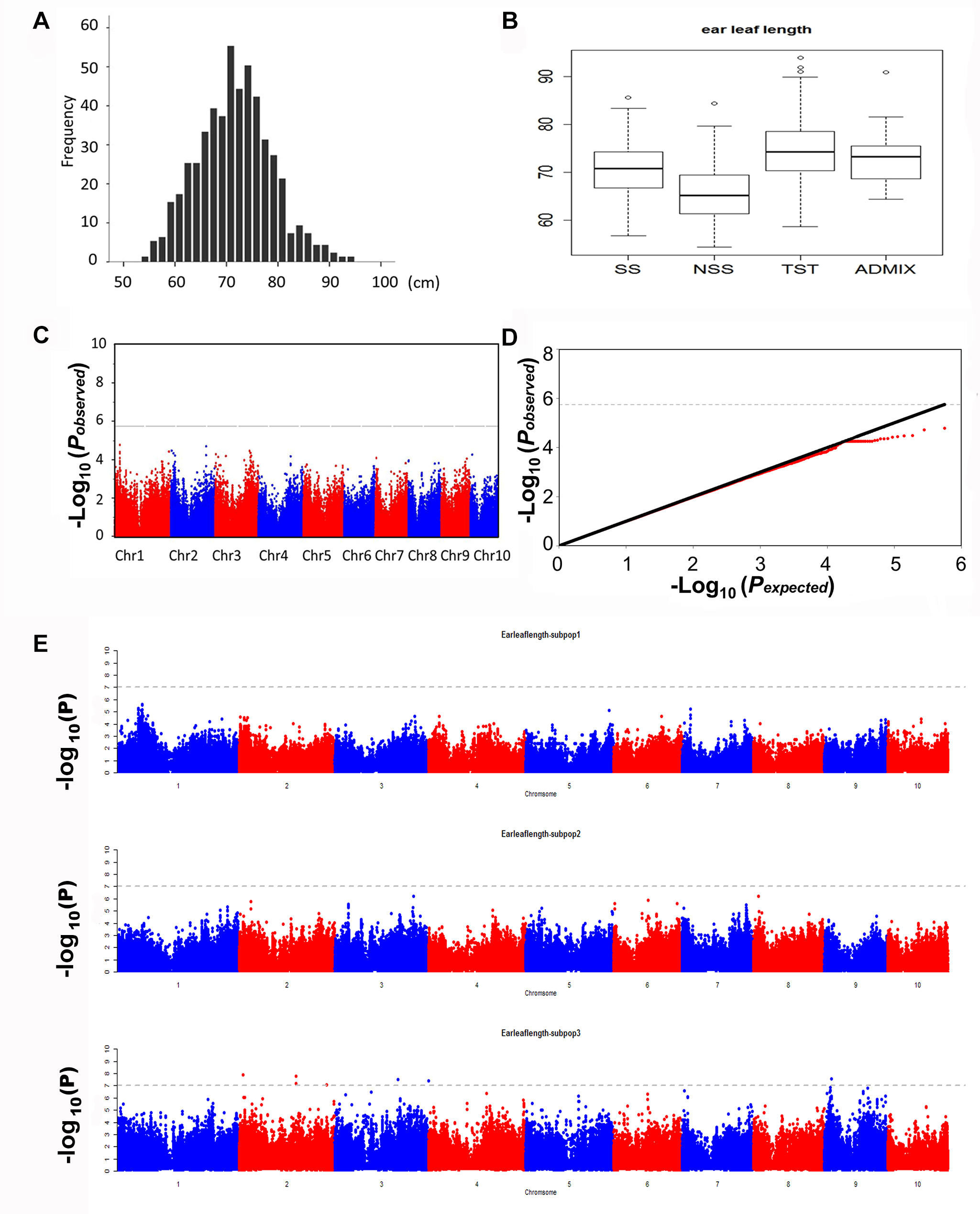

Supplement: Figure S4 — Genome-wide association analysis of ear leaf length. (A, B) Phenotype histogram and distribution of subpopulations in 513 maize lines. (C) Manhattan plots of mixed linear model conducted in imputation data, respectively. (D) Quantile-Quantile plots of p-values of mixed linear model conducted in imputation data. (E) Summary of GWAS results from Anderson-Darling test performed on each subpopulation independently for ear leaf length. (TIF) [file pgen.1004573.s004.tif]

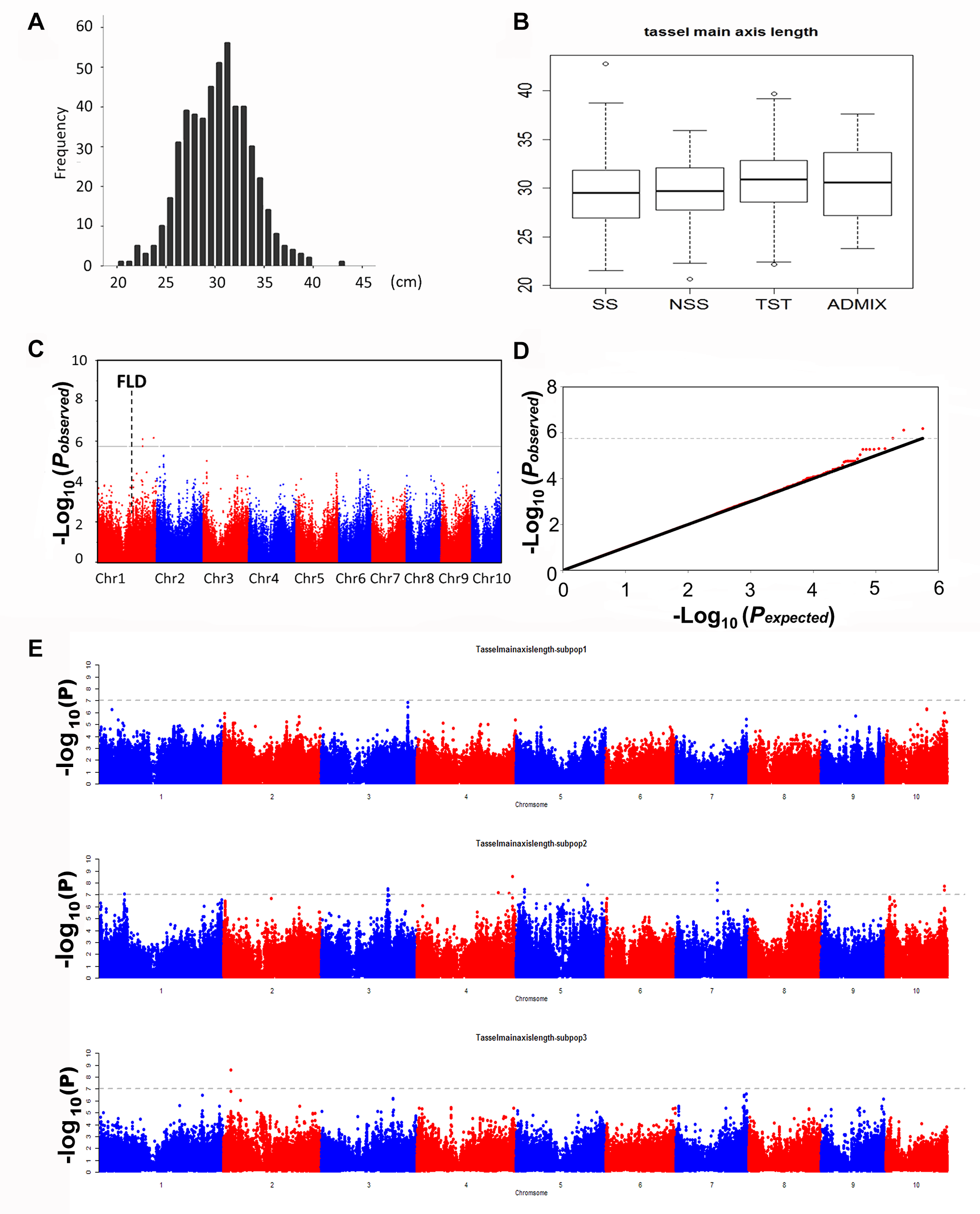

Supplement: Figure S5 — Genome-wide association analysis of tassel main axis length. (A, B) Phenotype histogram and distribution of subpopulations in 513 maize lines. (C) Manhattan plots of mixed linear model conducted in imputation data, respectively. (D) Quantile-Quantile plots of p-values of mixed linear model conducted in imputation data. Know genes controlling the traits were labeled. (E) Summary of GWAS results from Anderson-Darling test performed on each subpopulation independently for tassel main axis length. (TIF) [file pgen.1004573.s005.tif]

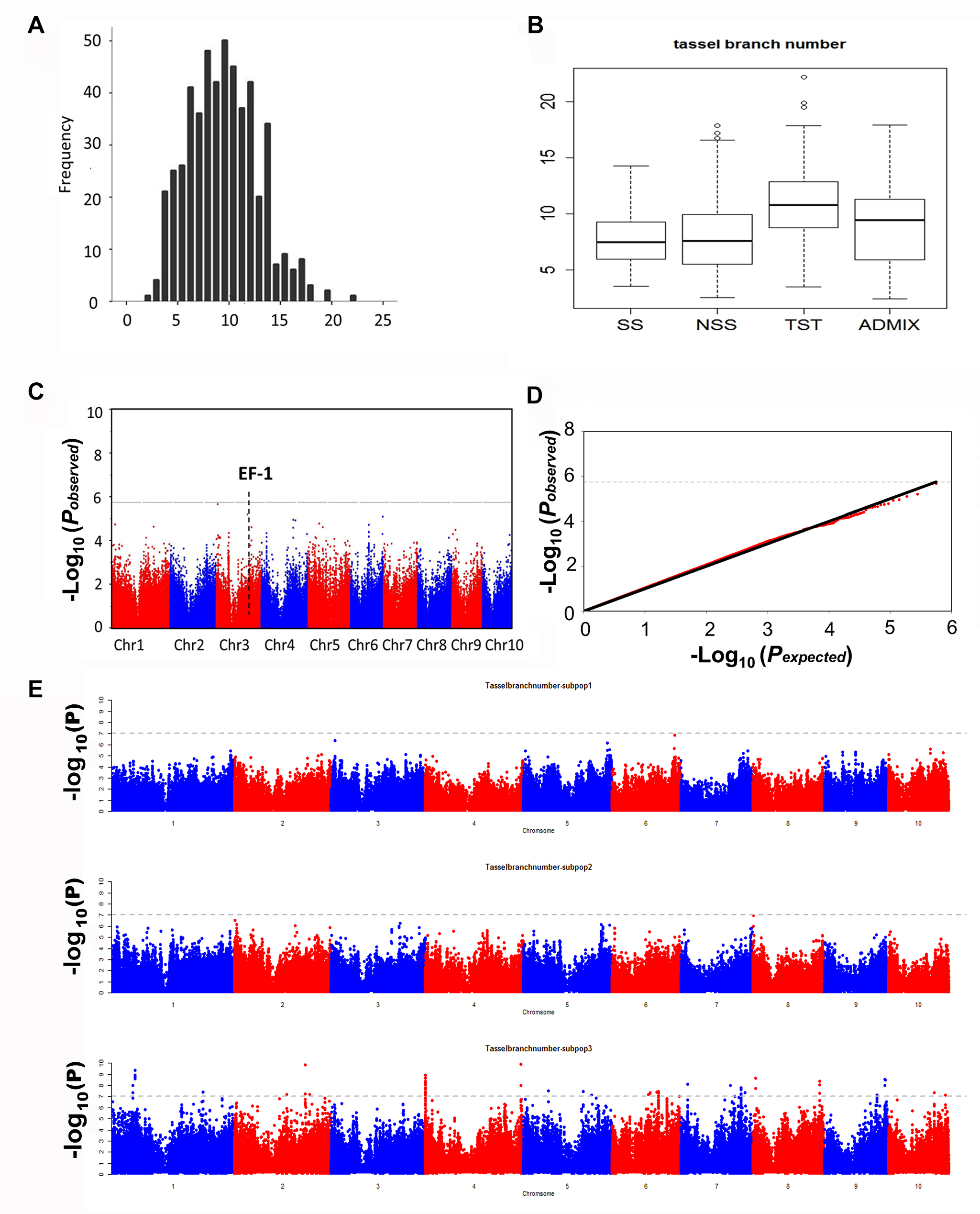

Supplement: Figure S6 — Genome-wide association analysis of tassel branch number. (A, B) Phenotype histogram and distribution of subpopulations in 513 maize lines. (C) Manhattan plots of mixed linear model conducted in imputation data, respectively. (D) Quantile-Quantile plots of p-values of mixed linear model conducted in imputation data. Know genes controlling the traits were labeled. (E) Summary of GWAS results from Anderson-Darling test performed on each subpopulation independently for tassel branch number. (TIF) [file pgen.1004573.s006.tif]

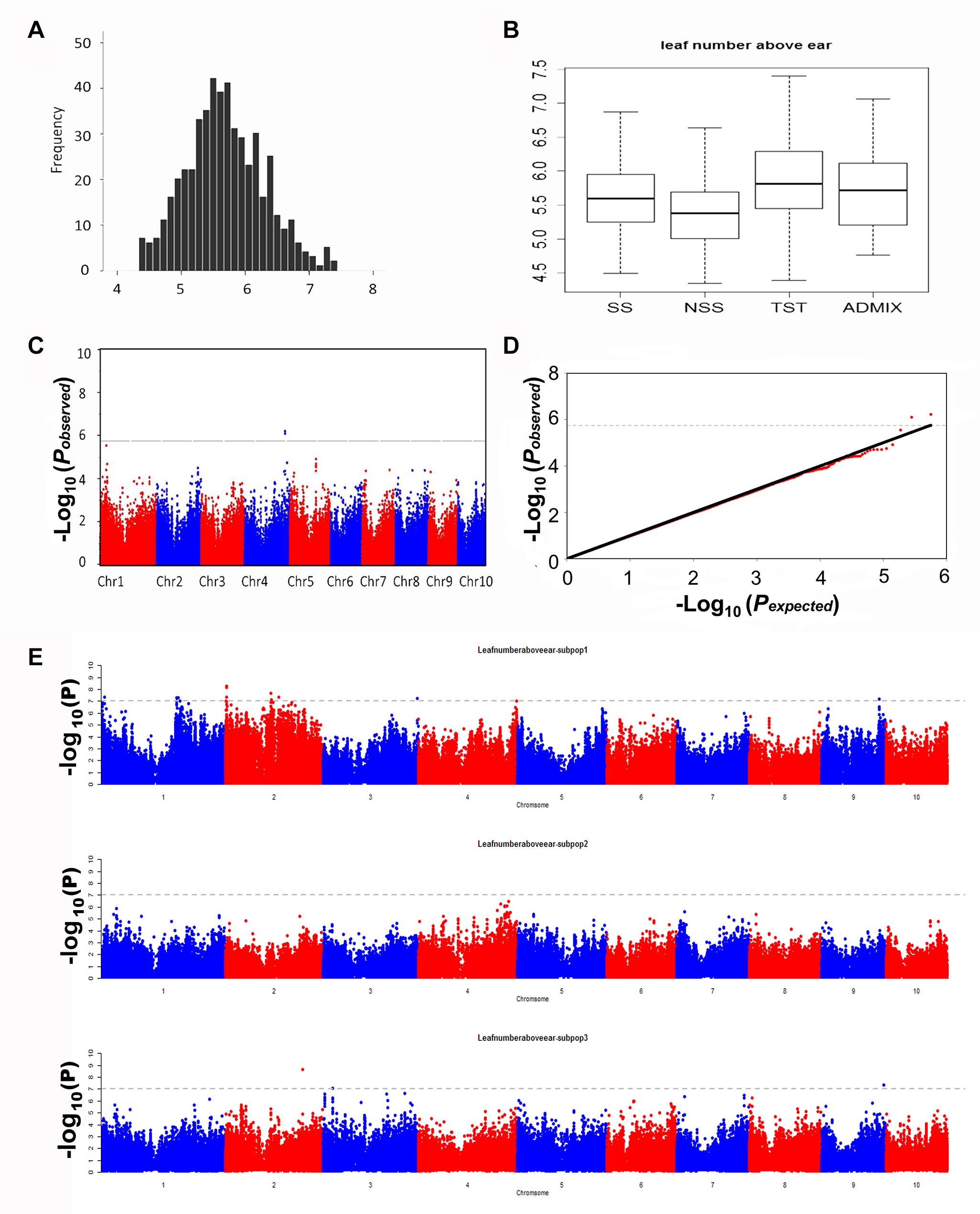

Supplement: Figure S7 — Genome-wide association analysis of leaf number above ear. (A, B) Phenotype histogram and distribution of subpopulations in 513 maize lines. (C) Manhattan plots of mixed linear model conducted in imputation data, respectively. (D) Quantile-Quantile plots of p-values of mixed linear model conducted in imputation data. (E) Summary of GWAS results from Anderson-Darling test performed on each subpopulation independently for leaf number above ear. (TIF) [file pgen.1004573.s007.tif]

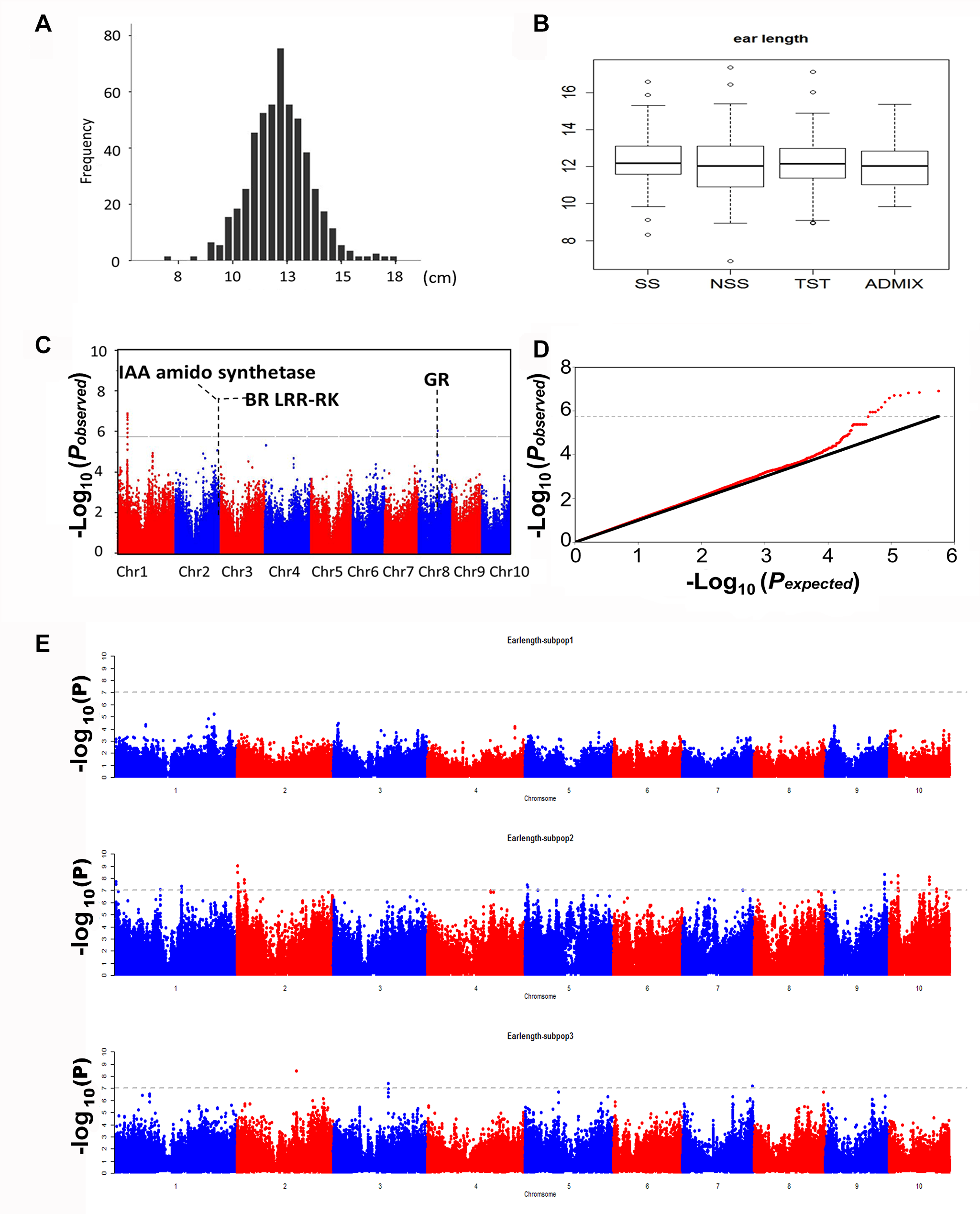

Supplement: Figure S8 — Genome-wide association analysis of ear length. (A, B) Phenotype histogram and distribution of subpopulations in 513 maize lines. (C) Manhattan plots of mixed linear model conducted in imputation data, respectively. (D) Quantile-Quantile plots of p-values of mixed linear model conducted in imputation data. Know genes controlling the traits were labeled. (E) Summary of GWAS results from Anderson-Darling test performed on each subpopulation independently for ear length. (TIF) [file pgen.1004573.s008.tif]

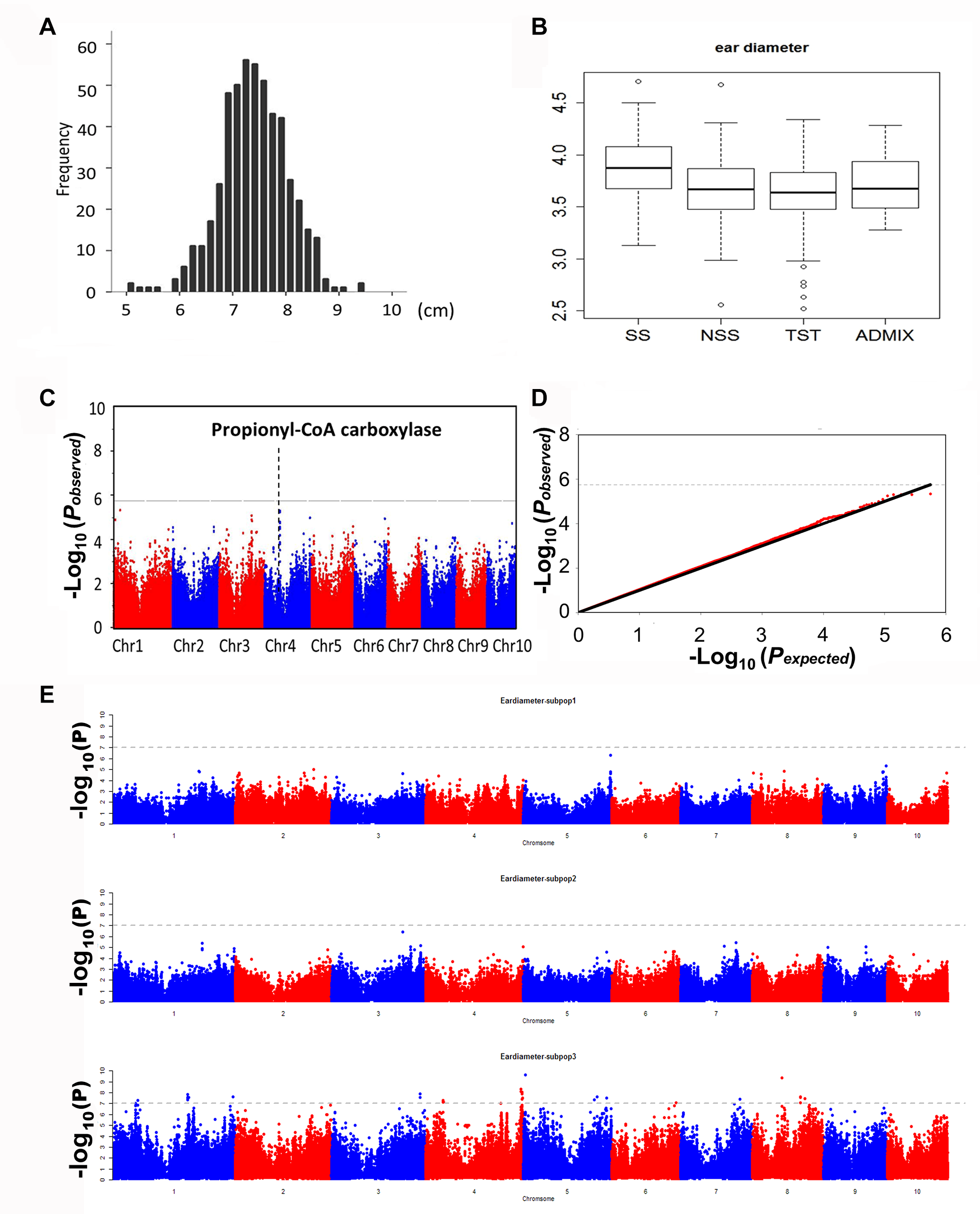

Supplement: Figure S9 — Genome-wide association analysis of ear diameter. (A, B) Phenotype histogram and distribution of subpopulations in 513 maize lines. (C) Manhattan plots of mixed linear model conducted in imputation data, respectively. (D) Quantile-Quantile plots of p-values of mixed linear model conducted in imputation data. Know genes controlling the traits were labeled. (E) Summary of GWAS results from Anderson-Darling test performed on each subpopulation independently for ear diameter. (TIF) [file pgen.1004573.s009.tif]

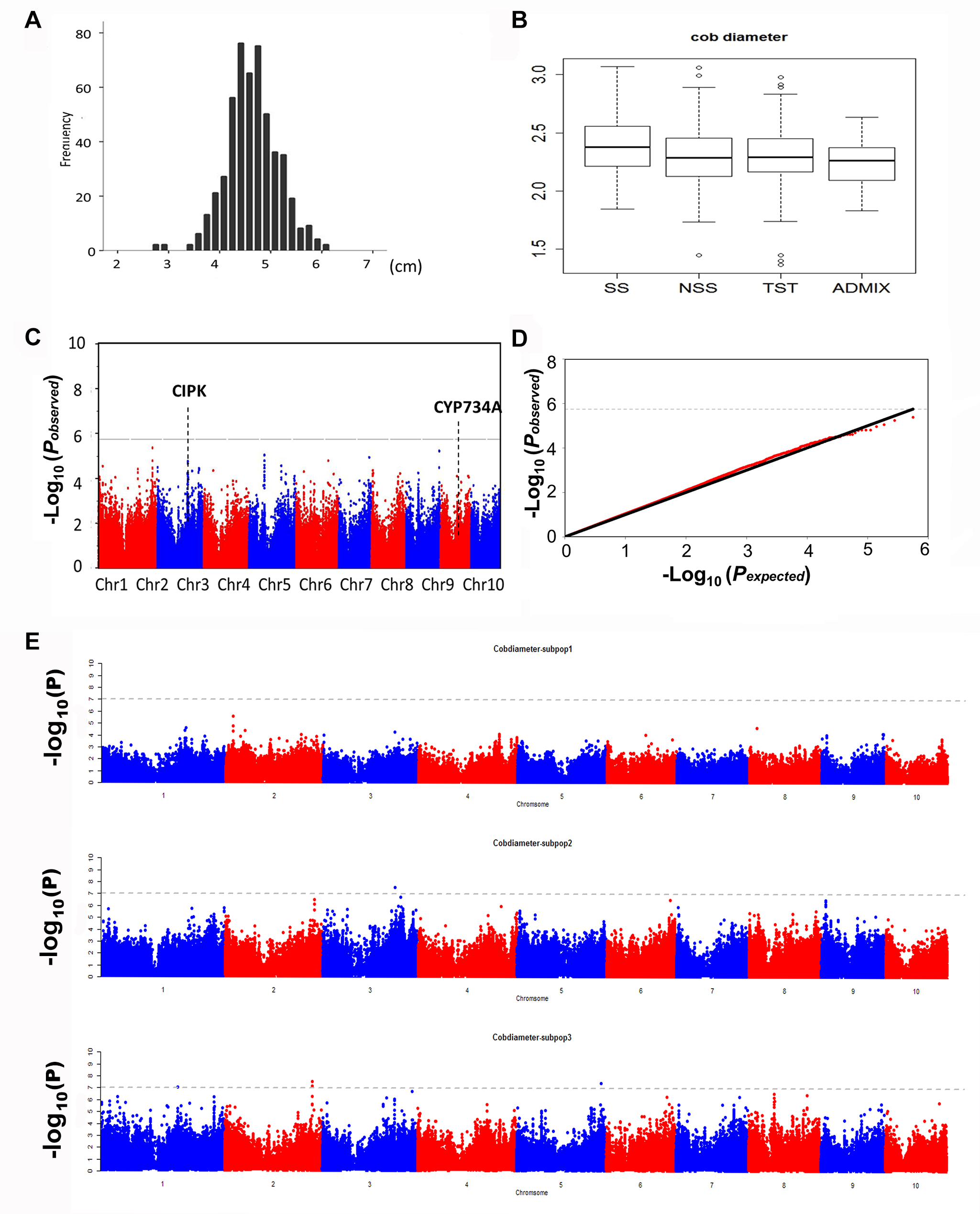

Supplement: Figure S10 — Genome-wide association analysis of cob diameter. (A, B) Phenotype histogram and distribution of subpopulations in 513 maize lines. (C) Manhattan plots of mixed linear model conducted in imputation data, respectively. (D) Quantile-Quantile plots of p-values of mixed linear model conducted in imputation data. Know genes controlling the traits were labeled. (E) Summary of GWAS results from Anderson-Darling test performed on each subpopulation independently for cob diameter. (TIF) [file pgen.1004573.s010.tif]

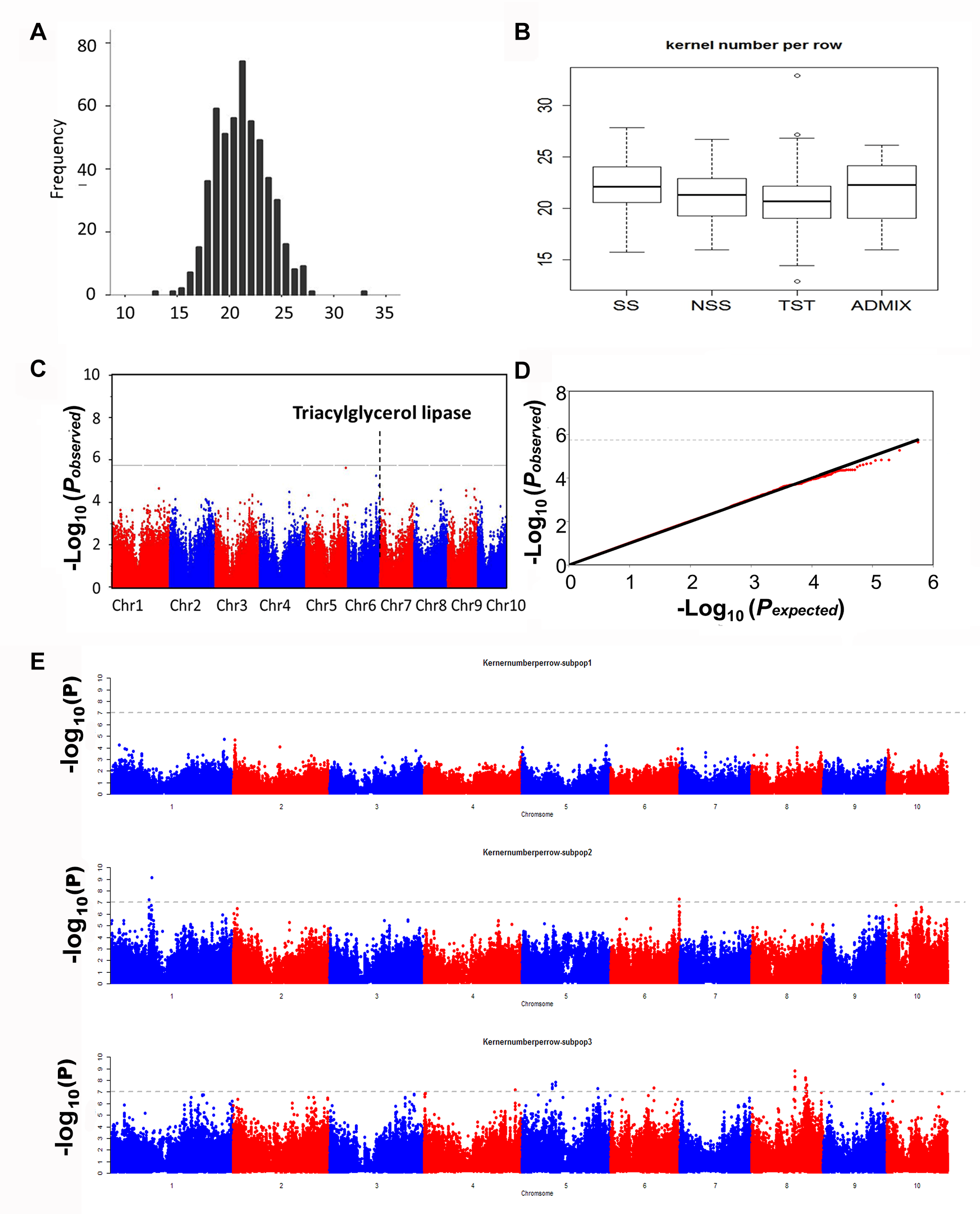

Supplement: Figure S11 — Genome-wide association analysis of kernel number per row. (A, B) Phenotype histogram and distribution of subpopulations in 513 maize lines. (C) Manhattan plots of mixed linear model conducted in imputation data, respectively. (D) Quantile-Quantile plots of p-values of mixed linear model conducted in imputation data. Know genes controlling the traits were labeled. (E) Summary of GWAS results from Anderson-Darling test performed on each subpopulation independently for kernel number per row. (TIF) [file pgen.1004573.s011.tif]

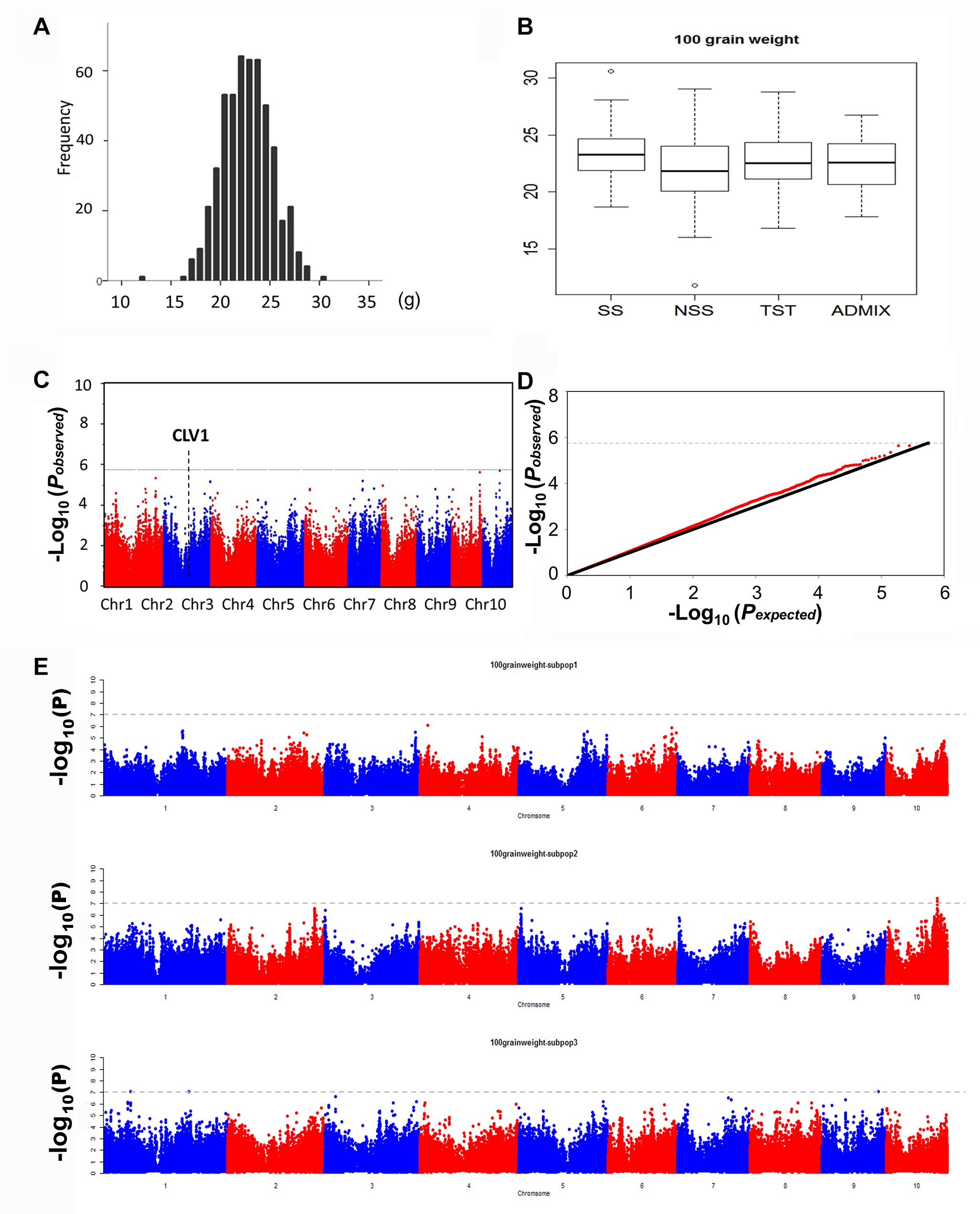

Supplement: Figure S12 — Genome-wide association analysis of 100-grain weight. (A, B) Phenotype histogram and distribution of subpopulations in 513 maize lines. (C) Manhattan plots of mixed linear model conducted in imputation data, respectively. (D) Quantile-Quantile plots of p-values of mixed linear model conducted in imputation data. Know genes controlling the traits were labeled. (E) Summary of GWAS results from Anderson-Darling test performed on each subpopulation independently for 100-grain weight. (TIF) [file pgen.1004573.s012.tif]

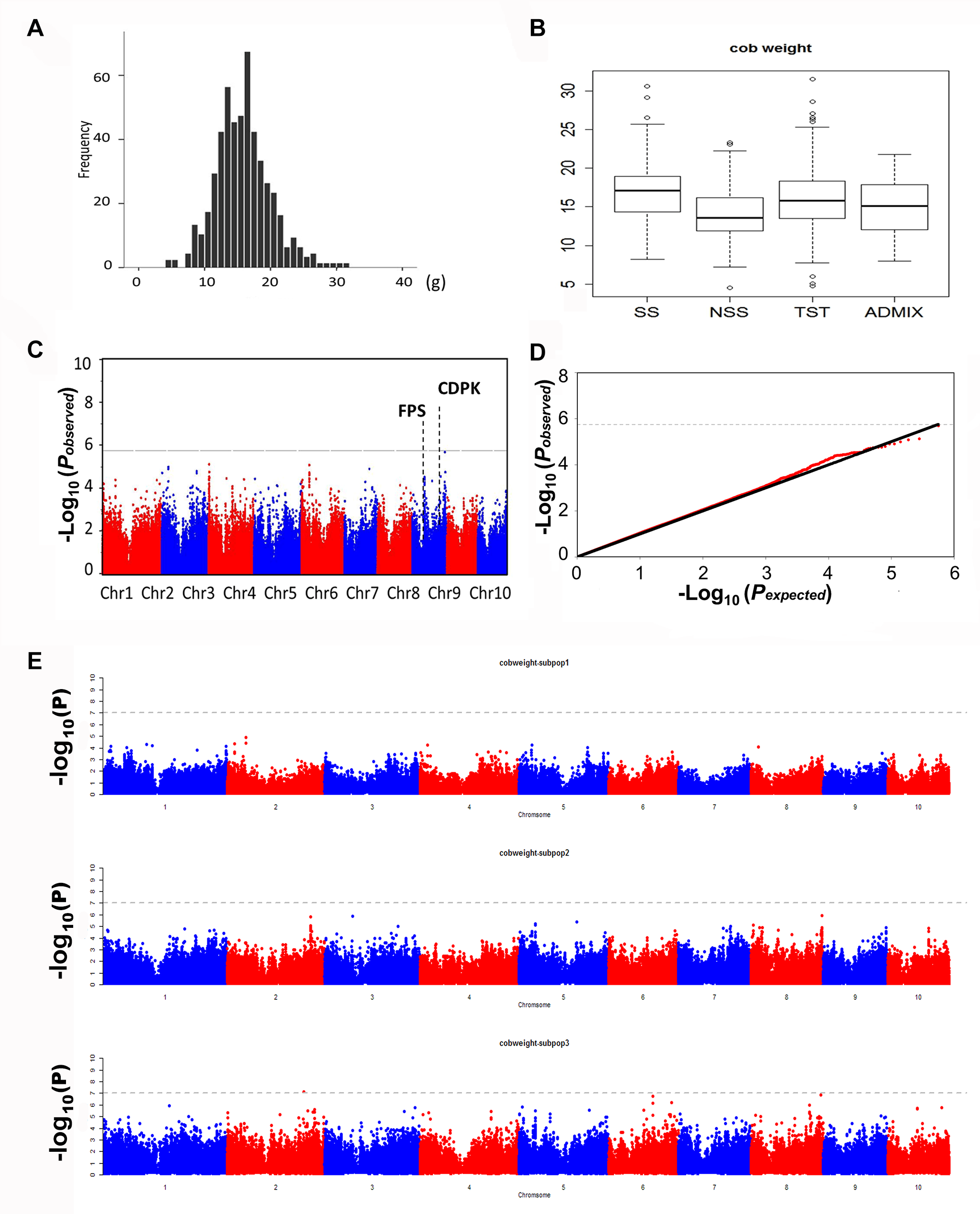

Supplement: Figure S13 — Genome-wide association analysis of cob weight. (A, B) Phenotype histogram and distribution of subpopulations in 513 maize lines. (C) Manhattan plots of mixed linear model conducted in imputation data, respectively. (D) Quantile-Quantile plots of p-values of mixed linear model conducted in imputation data. Know genes controlling the traits were labeled. (E) Summary of GWAS results from Anderson-Darling test performed on each subpopulation independently for cob weight. (TIF) [file pgen.1004573.s013.tif]

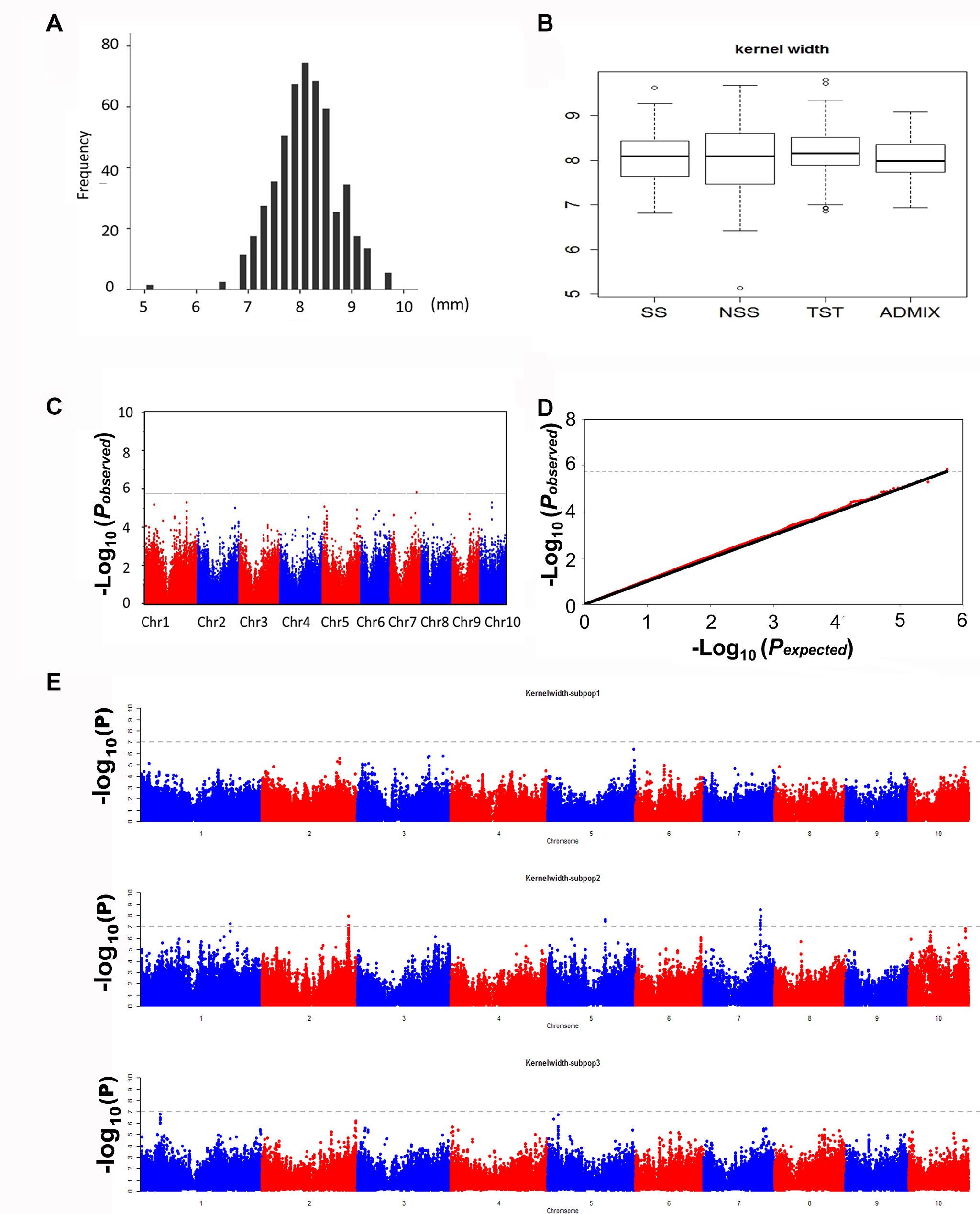

Supplement: Figure S14 — Genome-wide association analysis of kernel width. (A, B) Phenotype histogram and distribution of subpopulations in 513 maize lines. (C) Manhattan plots of mixed linear model conducted in imputation data, respectively. (D) Quantile-Quantile plots of p-values of mixed linear model conducted in imputation data. (E) Summary of GWAS results from Anderson-Darling test performed on each subpopulation independently for kernel width. (TIF) [file pgen.1004573.s014.tif]

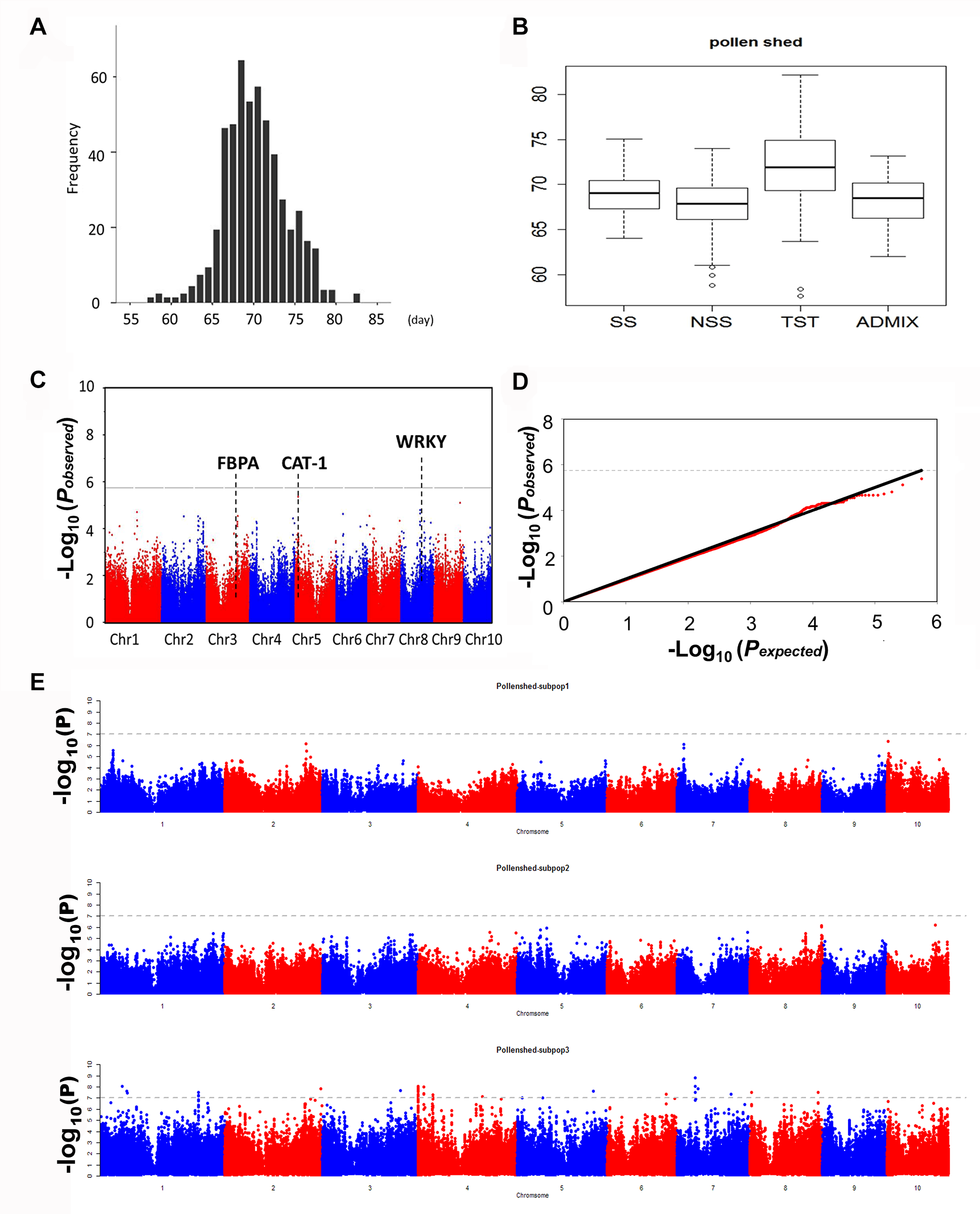

Supplement: Figure S15 — Genome-wide association analysis of days to anthesis. (A, B) Phenotype histogram and distribution of subpopulations in 513 maize lines. (C) Manhattan plots of mixed linear model conducted in imputation data, respectively. (D) Quantile-Quantile plots of p-values of mixed linear model conducted in imputation data. Know genes controlling the traits were labeled. (E) Summary of GWAS results from Anderson-Darling test performed on each subpopulation independently for days to anthesis. (TIF) [file pgen.1004573.s015.tif]

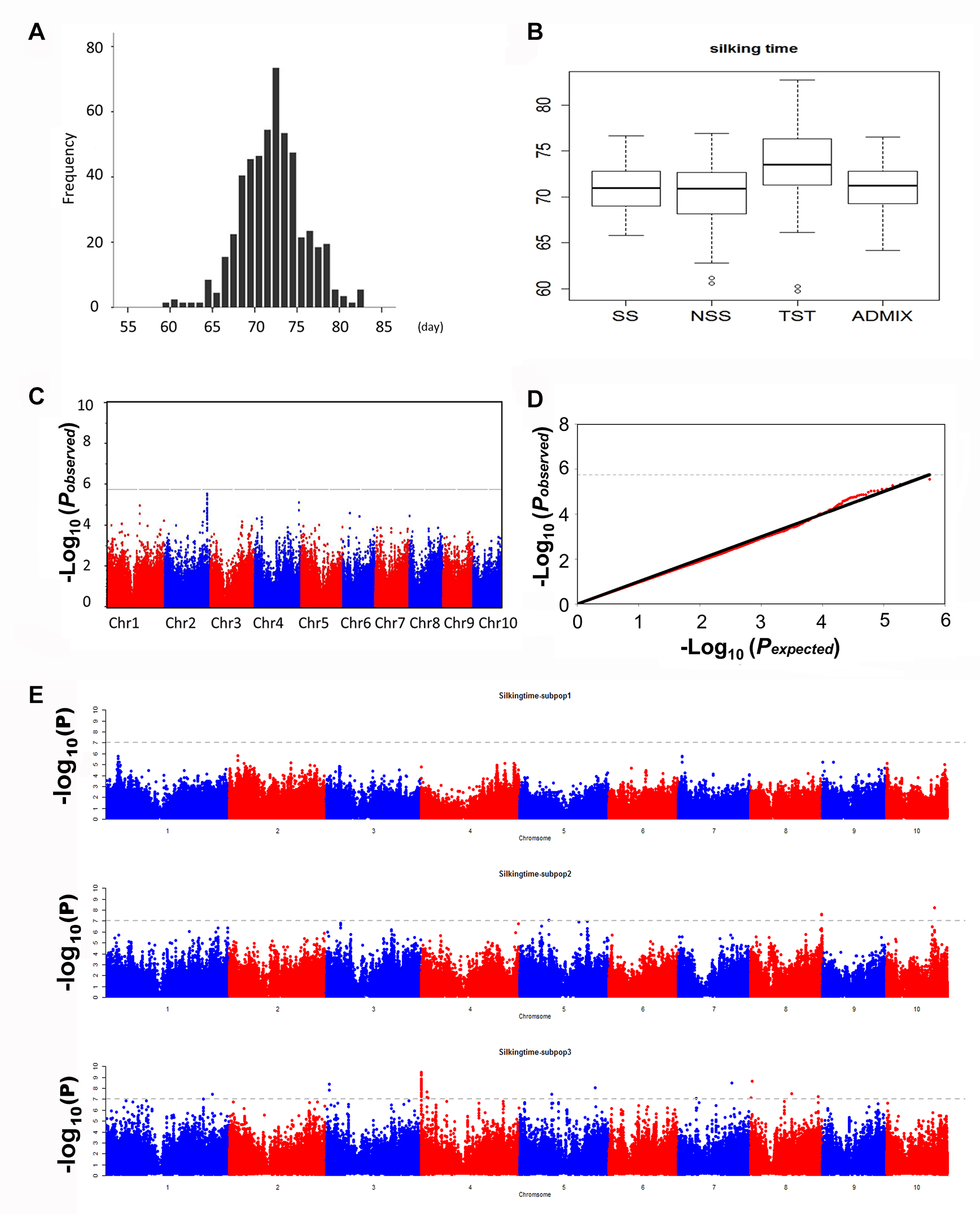

Supplement: Figure S16 — Genome-wide association analysis of days to silking. (A, B) Phenotype histogram and distribution of subpopulations in 513 maize lines. (C) Manhattan plots of mixed linear model conducted in imputation data, respectively. (D) Quantile-Quantile plots of p-values of mixed linear model conducted in imputation data. (E) Summary of GWAS results from Anderson-Darling test performed on each subpopulation independently for days to silking. (TIF) [file pgen.1004573.s016.tif]

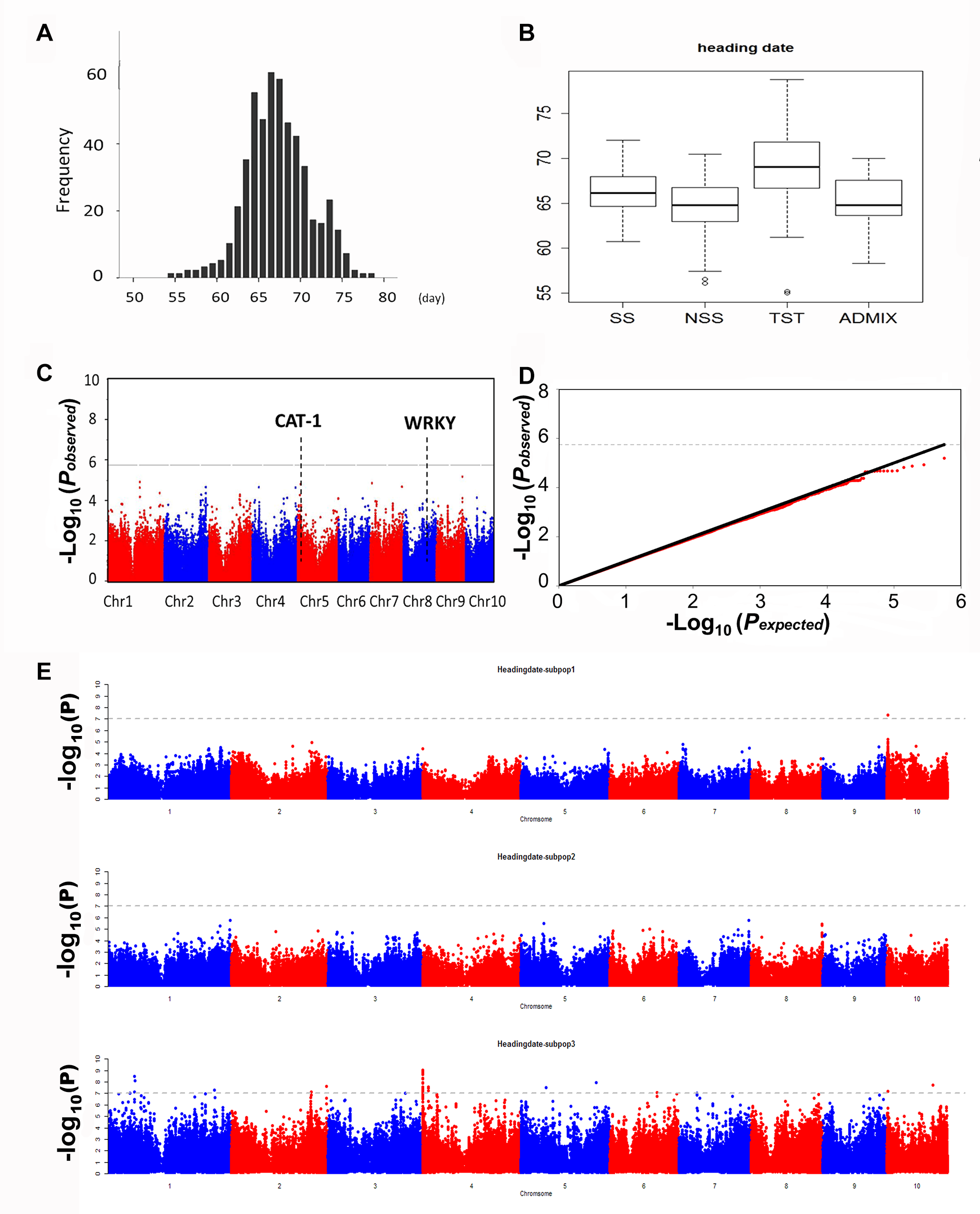

Supplement: Figure S17 — Genome-wide association analysis of days to heading. (A, B) Phenotype histogram and distribution of subpopulations in 513 maize lines. (C) Manhattan plots of mixed linear model conducted in imputation data, respectively. (D) Quantile-Quantile plots of p-values of mixed linear model conducted in imputation data. Know genes controlling the traits were labeled. (E) Summary of GWAS results from Anderson-Darling test performed on each subpopulation independently for days to heading. (TIF) [file pgen.1004573.s017.tif]

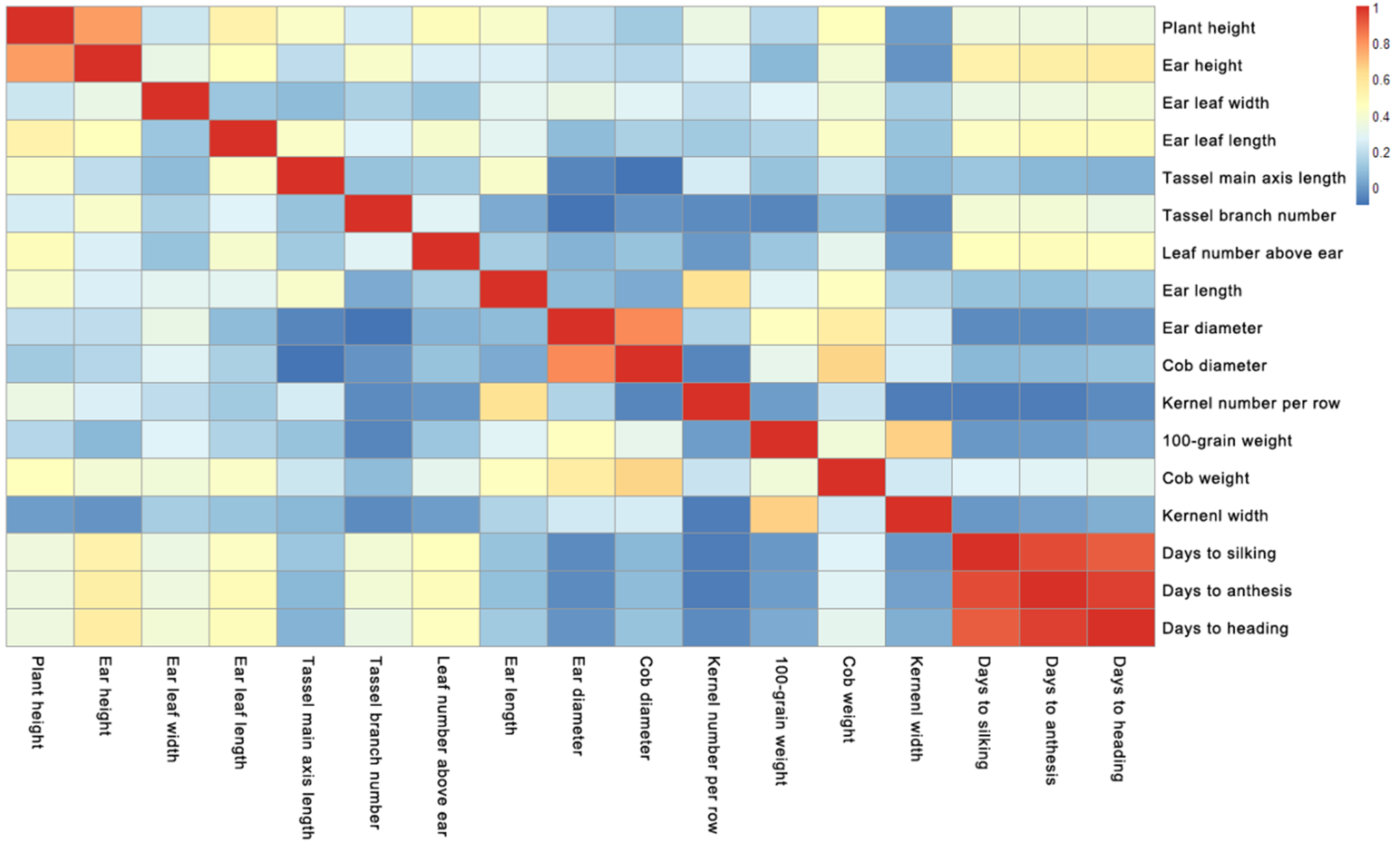

Supplement: Figure S18 — Pair-wise Pearson's correlation among 17 traits in 513 maize lines. (TIF) [file pgen.1004573.s018.tif]

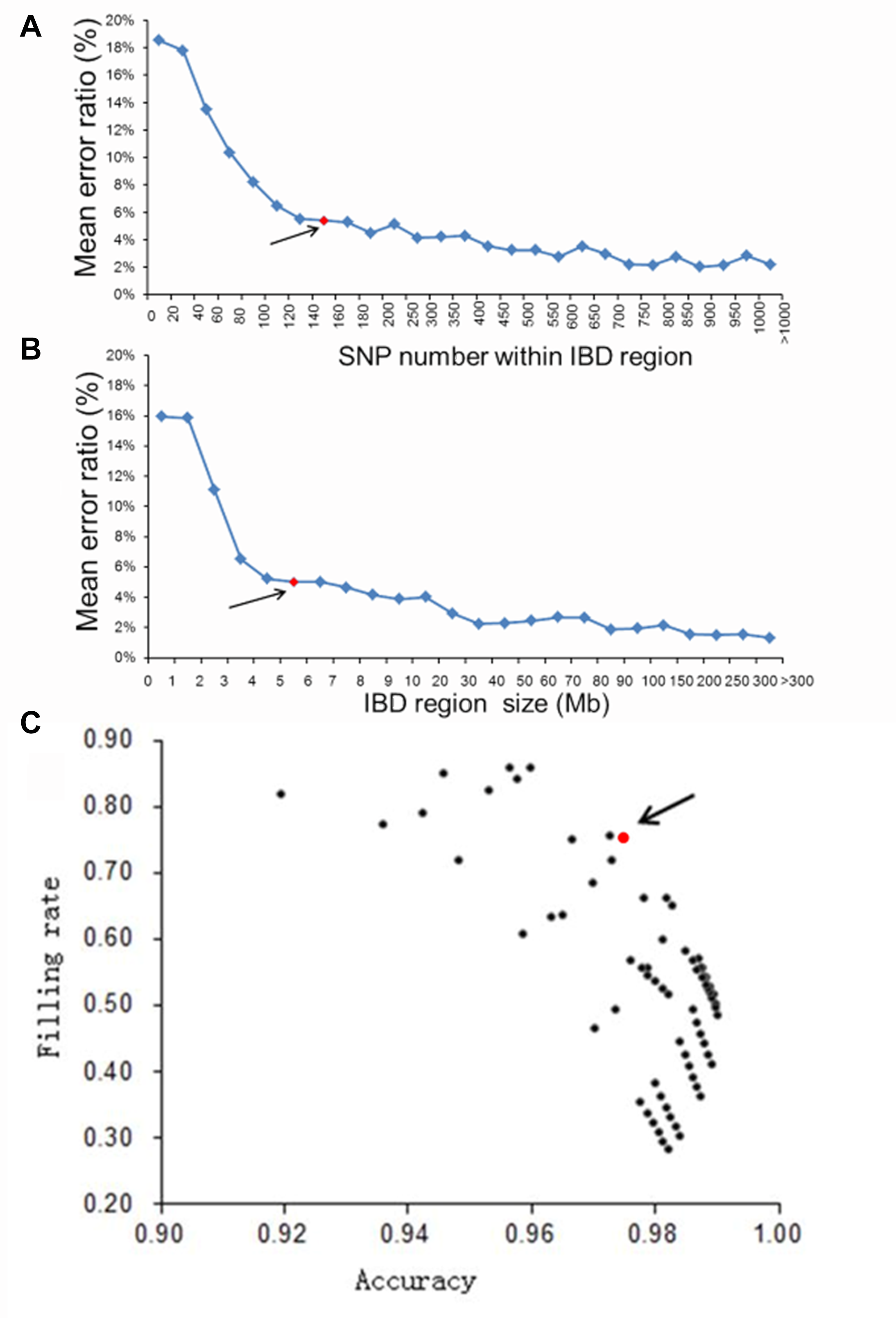

Supplement: Figure S19 — The mean error ratio and mean coverage ratio pool over SNP number within IBD region (A) and size of IBD region (B), respectively, on chromosome 1 for the 368 maize lines. (C) Imputation accuracy and filling rate for each of 72 combinations of variables of KNN. The combination, indicated by arrow, was chosen for final data imputation. (TIF) [file pgen.1004573.s019.tif]
